# Water‐Triggered Reconfigurable Glycerogels for Sustainable All‐Gel Supercapacitors

**DOI:** 10.1002/advs.202411847

**Published:** 2024-12-04

**Authors:** Md. Tariful Islam Mredha, Adith Varma Rama Varma, Tanish Gupta, Insu Jeon

**Affiliations:** ^1^ School of Mechanical Engineering Chonnam National University 77 Yongbong‐ro, Buk‐gu Gwangju 61186 Republic of Korea

**Keywords:** all‐gel supercapacitors, biopolymer glycerogels, degradable glycerogels, reconfigurable glycerogels, reversible crosslinks, self‐healing, wide temperature stability

## Abstract

The polymeric structures of synthetic gels are typically static, which makes them damage‐prone and nonrecyclable. Inspired by the dynamic reconfigurability of biological tissues, which eliminate old/damaged cells and regenerate new ones via biological triggers/signals, a reconfigurable biopolymer gel is presented based on a glycerol‐mediated supramolecular gelation strategy. In response to an eco‐friendly triggering agent water, this gel undergoes on‐demand molecular‐level reconfiguration. The versatility of the approach enables the development of reconfigurable gels with modulated functionality. As a proof‐of‐concept, a reconfigurable glycerogel electrode and electrolyte are developed and used to prototype an all‐gel supercapacitor that exhibits exceptional self‐healing, degradation, and rebuilding abilities. Furthermore, it can tolerate extreme mechanical deformations (e.g., stretching, bending, and twisting) and temperatures (−20 to 80 °C). The device exhibits excellent energy storage performance, with a maximum areal capacitance of 450 mF cm^−2^ (at 0.035 mA cm^−2^) and remarkable capacitance retention of 89% following 20 000 charge/discharge cycles (at 0.35 mA cm^−2^). Moreover, following self‐healing and rebuilding, the capacitance remains at 91% and 110% (at 0.35 mA cm^−2^) of the original value, respectively. This generalized strategy for preparing multifunctional reconfigurable gels will facilitate the development of high‐performance flexible and wearable devices with improved durability and recyclability.

## Graphical abstract

In this study, a bioinspired glycerol‐mediated supramolecular gelation strategy is developed for the synthesis of biopolymer gels with reconfigurable cross‐linking structures. In contrast to conventional synthetic gels, these gels exhibited on‐demand comprehensive reconfiguration abilities—ranging from macro to molecular scales—simply via water‐triggering. Using this strategy, a sustainable all‐gel supercapacitor with remarkable self‐healing, degradation, and rebuilding capabilities is prepared.

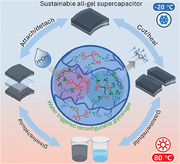

## 1 Introduction

Biological tissues constantly change and remodel to achieve adaptability and longevity.^[^ ^1^, ^2^, ^3^ ^]^ Conversely, synthetic gels typically lack the capacity for tissue‐like reconstruction despite their biomimetic structures and functionalities.^[^ ^1^, ^2^, ^3^, ^4^ ^]^ This is because their polymeric structures are inherently static, preventing them from undergoing molecular‐level reconfiguration.^[^ ^1^, ^2^ ^]^ In recent years, gel utilization has increased considerably in fields such as soft robotics and stretchable and wearable devices.^[^ ^4^, ^5^, ^6^, ^7^, ^8^ ^]^ More than a billion individuals across the globe already use wearable devices,^[^ ^9^ ^]^ and the global market for smart wearables, valued at USD 60 billion in 2023, is forecast to increase to USD 375 billion by 2033.^[^ ^10^ ^]^ This is likely to result in considerable environmental waste if synthetic gels that lack adaptive and reconstructive abilities are used, necessitating immediate, innovative, and sustainable management solutions.^[^ ^11^, ^12^, ^13^ ^]^

Compared with conventional solid or solid/liquid supercapacitors, all‐gel supercapacitors (AGSCs) offer unique advantages for stretchable and wearable devices.^[^ ^14^, ^15^ ^]^ However, AGSCs are still in the early stages of development and face several notable challenges that hinder their practical application.^[^ ^14^, ^15^, ^16^, ^17^, ^18^, ^19^, ^20^, ^21^, ^22^, ^23^ ^]^ First, most reported AGSCs are constructed from non‐degradable, environmentally unfriendly, and non‐biocompatible materials.^[^ ^14^, ^15^, ^16^, ^17^, ^18^, ^19^ ^]^ Second, AGSC fabrication strategies typically involve the self‐healing of predesigned gel components (electrodes and electrolytes) with electrical and ionic conductivity.^[^ ^14^, ^15^, ^16^ ^]^ Nevertheless, the structural and mechanical incompatibility between the predesigned components often leads to weak interfaces and poor electrochemical performance, which is exacerbated by deformation. An all‐in‐one fabrication strategy has also been developed, whereby electroactive layers are created in situ on the surface of electrolyte gels to form electrodes.^[^ ^17^, ^18^, ^19^, ^20^, ^21^ ^]^ However, the electrode layers are typically brittle with low electrical conductivity, thereby yielding poor mechanical stability and energy loss. Third, reported AGSCs are primarily composed of water‐borne hydrogels or organohydrogels, which are environmentally unstable;^[^ ^12^, ^13^, ^14^, ^15^, ^16^, ^17^, ^18^, ^19^, ^20^, ^21^, ^22^, ^23^ ^]^ the in‐air performance is significantly hindered by the natural evaporation of water. Finally, most reported AGSCs cannot undergo structural reconfiguration, although some self‐healable AGSC components do exhibit reconfigurability.^[^ ^11^, ^12^, ^13^, ^14^, ^15^, ^16^, ^17^, ^18^, ^19^, ^20^, ^21^, ^22^, ^23^ ^]^ Single‐use AGSCs will result in a significant amount of electronic waste. AGSCs show considerable promise in advancing flexible and wearable devices, health‐monitoring sensors, and point‐of‐care diagnostics. However, no specific strategy has been reported for developing fully reconfigurable, high‐performance AGSCs. To ensure the sustainable long‐term use of AGSCs in wearable and stretchable devices, the aforementioned problems need to be addressed.

**Figure 1:**
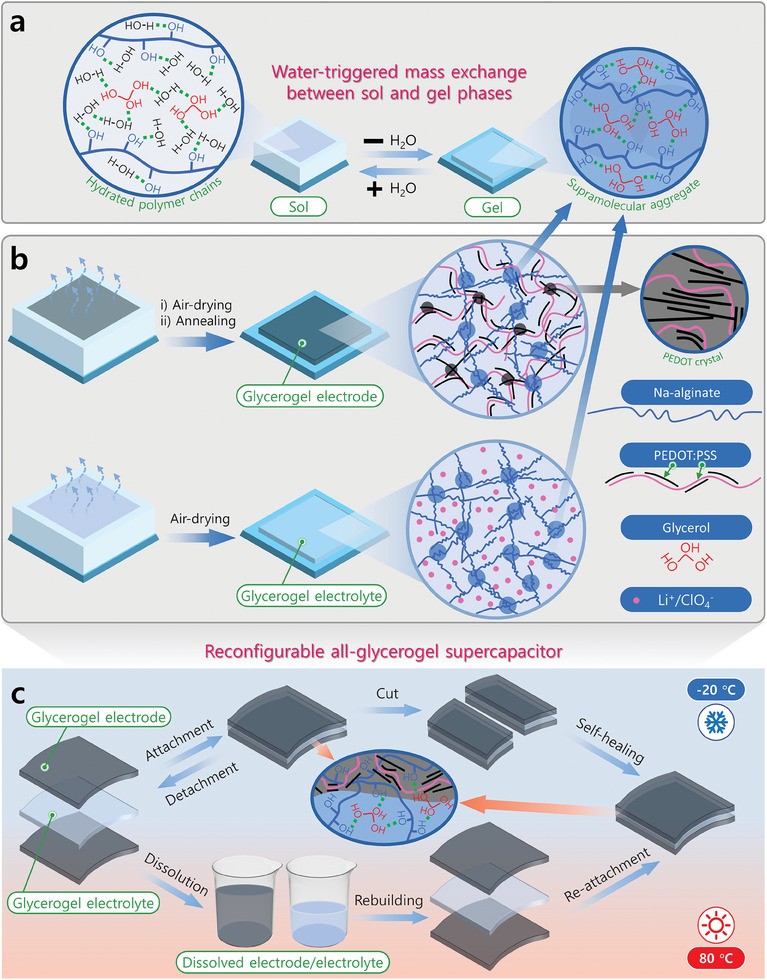
The design strategy of reconfigurable glycerogels. a) Illustration of a reconfigurable gel undergoing facile and reversible mass exchange between the sol and gel states by water‐triggering. In the presence of water, the polymer and glycerol are in a hydrated solution state. Upon the removal of water by evaporation, the system transitions to glycerogel. Glycerol acts as a supramolecular gelling agent owing to its strong hydrogen‐bonding ability with polysaccharides, providing the glycerogels with strong yet switchable supramolecular architectures. b) Fabrication strategy of reconfigurable glycerogels. Precursor solutions are air‐dried to form reconfigurable glycerogel electrodes and electrolytes. Annealing of the glycerogel electrode facilitates PEDOT:PSS crystallization, which enhances performance without losing reconfigurability. c) A reconfigurable, high‐performance AGSC is prepared from glycerogel electrodes and electrolyte. The AGSC exhibits self‐healing and rebuilding abilities and can tolerate extreme temperatures (−20 to 80 °C).

In this study, we present a bioinspired and environmentally friendly strategy for developing multifunctional glycerogels and glycerogel‐based devices that can be reconfigured at the molecular level. This strategy was inspired by the remodeling ability of biological tissues, which undergo mass exchange between their solid (e.g., biopolymers and cells) and liquid (e.g., water, small ions, and molecules) phases to destruct old cells and reconstruct new ones.^[^ ^1^, ^24^, ^25^ ^]^ This process is often triggered by biosynthesized molecules. For instance, in bone remodeling, osteoclasts secrete acid and collagenase to dissolve bone, whereas osteoblasts secrete collagen that is physically crosslinked with bone minerals for reconstruction.^[^ ^25^, ^26^ ^]^ In a similar way, our biopolymer‐based glycerogel system exhibits facile mass exchange between its solid and liquid phases, which enables supramolecular reconfiguration in response to water (**Figure** **1**a). These water‐triggered glycerogels are structurally reconfigurable and adaptable to different environments. Moreover, they can be easily modulated to achieve a wide range of functionalities.

We prepared electrically and ionically conductive reconfigurable glycerogels to prototype a sustainable, high‐performance AGSC (Figure 1b,c). The fabricated AGSC demonstrated a maximum areal capacitance of ≈450 mF cm^−2^ (at a current density of 0.035 mA cm^−2^) and a capacitance retention of ≈89% after 20 000 charge/discharge cycles (at a current density of 0.35 mA cm^−2^). Furthermore, the device tolerated extreme temperatures (ranging from −20 to 80 °C) and mechanical deformations (stretching, bending, and twisting). Owing to its unique water‐triggered molecular reconfiguration capability, the developed AGSC demonstrated excellent self‐healing, degradation, and rebuilding properties. The concept presented in this study is anticipated to pave the way for developing diverse, high‐performance, and reconfigurable gels and gel‐based devices by utilizing different combinations of polymers and functional materials. They could be sustainably used in next‐generation flexible and wearable devices.

## 2 Results and Discussion

### 2.1 Design Strategy of Reconfigurable Glycerogels

Na‐alginate, a widely used biocompatible and biodegradable polysaccharide,^[^ ^4^, ^27^ ^]^ was employed as the model system to demonstrate our reconfigurable gels. Most polysaccharides form stable gels through supramolecular bonding. Because of the rigid nature of linear polysaccharides, they generate internal stress during supramolecular interactions, thereby facilitating the formation of microscopic domains with an aggregated structure. However, when water is used as the solvent, the typical supramolecular structures in alginate‐based hydrogels are formed through metal‐ion (Ca^2+^, Cu^2+^, Zn^2+^, Al^3+^, etc.)‐mediated ionic/coordination‐bonding and hydrogen‐bonding.^[^ ^27^, ^28^ ^]^ These structures are not fully retrievable to the original state (sol) because of the formation of some irreversible aggregates. Therefore, developing fully reconfigurable and stretchable gels from alginate/water systems is challenging.

Glycerol, an inexpensive and environmentally friendly bioderived compound, has been used as a solvent to prepare alginate gels with reconfigurable structures (Figure 1a). Moreover, the unique properties of glycerol, such as its exceptional supercoolability (as low as −83 °C), extremely low vapor pressure (133.3 Pa at 125 °C), and extremely high boiling temperature (290 °C) make the resultant gels tolerant to extreme environments.^[^ ^5^, ^14^, ^29^ ^]^ The additional hydroxyl groups and the bulkier structure of glycerol compared to that of pure water contribute to a more robust and stable hydrogen‐bonded structure with polysaccharides. Thus, glycerol functions as both a solvent and crosslinker in alginate/glycerol systems. Furthermore, glycerol has a lower polarity than water. Because of these features, the gel point of Na‐alginate is lower in glycerol than in water. For instance, a 10 wt.% Na‐alginate solution in glycerol exists as a stretchable gel (Figure S1a,b and Movie S1, Supporting Information), whereas the solution in water exists as a viscous slurry (Figure S1c,d and Movie S2, Supporting Information).

As such, glycerol is readily miscible with water via facile hydrogen bond formation. Therefore, the glycerol‐mediated supramolecular aggregates in alginate glycerogels, which are water‐soluble but glycerol‐insoluble, are expected to be switchable through water‐triggering (Figure 1a). That is, the addition of water would revert the gel state to a sol, while the gel state would form upon water evaporation. Notably, mass exchange between the gel and sol phases would enable self‐healing and reconfiguration capabilities. By controlling the polymer‐to‐solvent ratio, we can create reconfigurable supramolecular aggregates in an alginate‐based glycerogel system.

### 2.2 Fabrication of Reconfigurable Glycerogel Electrode and Electrolyte

Based on the rational design strategy described above, we developed reconfigurable alginate‐based glycerogels using a straightforward drying‐induced sol/gel process, as depicted in Figure 1. Electrically and ionically conductive reconfigurable glycerogels were fabricated to serve as the electrode and electrolyte, respectively, for a sustainable AGSC (Figure 1b).

The electrically conductive glycerogel electrode was prepared from a solution of Na‐alginate, poly(3,4‐ethylenedioxythiophene):poly(styrenesulfonate) (PEDOT:PSS), glycerol, and water. The incorporation of PEDOT:PSS was based on its biocompatibility, exceptional capacitance and electrical conductivity.^[^ ^6^, ^14^, ^30^, ^31^ ^]^ Furthermore, PEDOT:PSS is water‐dispersible and can form various aggregated structures depending on its processing.^[^ ^6^, ^14^, ^30^, ^31^ ^]^ The solution was cast into a rectangular mold and then air‐dried. As the water gradually evaporated, the polymer chains moved closer together, and once the water had completely evaporated, the polymer concentration in glycerol increased to its gel point. At this stage, glycerol facilitated the formation of aggregated, crosslinked alginate domains through hydrogen bonding, thereby providing mechanical integrity. Concurrently, the PEDOT:PSS formed an interpenetrating percolated structure with PEDOT crystals in the gel network. This structure provided electrical conductivity. The reconfiguration ability of the glycerogel was not affected by the incorporation of PEDOT:PSS because of its water‐dispersible nature.

The ionically conductive glycerogel electrolyte was fabricated from a solution of Na‐alginate, lithium perchlorate (LiClO_4_), glycerol, and water using a similar strategy (Figure 1b). The selection of LiClO_4_ was based on its high solubility in glycerol and extensive usage as an ion‐conducting material in energy storage devices. The hydrogen‐bonded alginate structure provided mechanical integrity, while the dissolved Li^+^ and ClO_4_^−^ ions in the gel phase provided ionic conductivity.

The most interesting feature of our fabrication strategy is the capacity to finely adjust the bonding strength. Glycerol acts as a triggerable molecular switch that can facilitate the formation and disruption of inter‐ and intra‐polymer interactions, giving it the capacity to self‐heal and rebuild. By precisely controlling the polymer density, we fabricated soft, stretchable, and reconfigurable glycerogel electrodes and electrolytes. These components could be readily assembled and reconfigured into any form, facilitating the development of a sustainable AGSC with a wide operating temperature range (Figure 1c).

### 2.3 Mechanical Properties and Environmental Tolerance of Reconfigurable Glycerogels

**Figure 2:**
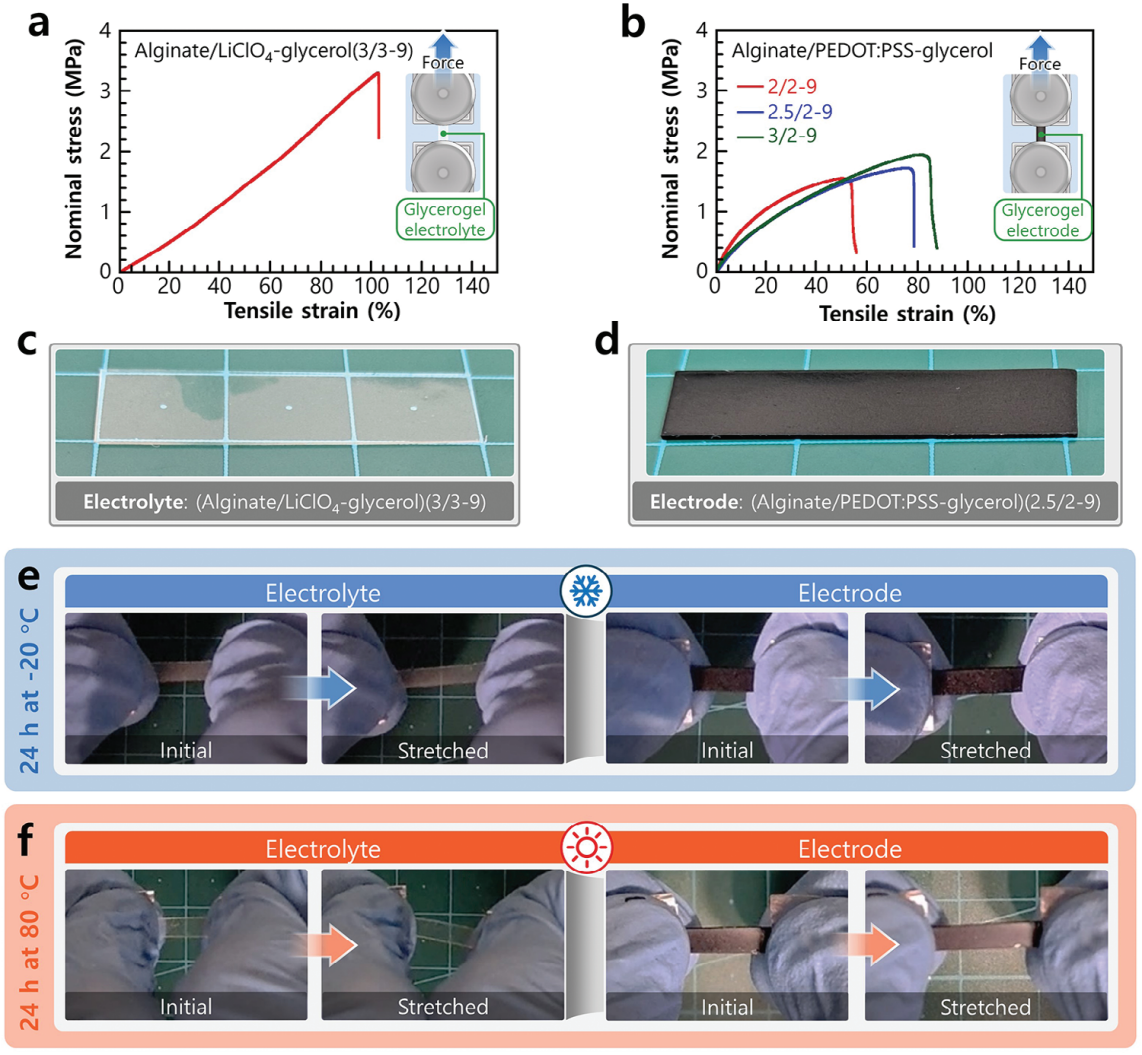
Mechanical performance of glycerogel electrolyte and electrode. Representative tensile stress–strain curves of the glycerogel a) electrolyte and b) electrode (insets: schematic of the tensile testing setup) and c,d) photographs of pristine glycerogel electrolyte and electrode. e,f) Demonstration of extreme temperature tolerance of the glycerogel electrolyte (left) and electrode (right) by stretching the gels after 24 h incubation at e) −20 °C and f) 80 °C.

The mechanical properties of the glycerogel electrolytes and electrodes were assessed under tensile testing (**Figure** **2**a,b). A typical glycerogel electrolyte denoted as alginate/LiClO_4_‐glycerol(3/3‐9), where the values in parentheses correspond to the concentrations of Na‐alginate (3 wt.%), LiClO_4_ (3 wt.%), and glycerol (9 wt.%) in the aqueous precursor solution, respectively. It exhibited an excellent tensile strength of 2.99 ± 0.41 MPa, elastic modulus of 2.31 ± 0.45 MPa, and stretchability (tensile strain) of 94% ± 8% (Figure 2a). In addition, it exhibited an excellent ionic conductivity of 0.41 ± 0.026 mS cm^−1^ (Figure S2, Supporting Information), which is of the same order as that of our recently reported LiClO_4_‐containing glycerogels.^[^ ^14^, ^29^ ^]^

To identify a mechanically compatible electrode, we designed glycerogels with different concentrations of alginate and PEDOT:PSS. As the alginate‐to‐PEDOT:PSS ratio increased, the strength and stretchability of the electrode increased, whereas its modulus decreased (Figure 2b; Figure S3, Supporting Information). Increasing the alginate concentration increased the ratio of hydrogen‐bonded alginate aggregates to PEDOT crystals. This increased the toughness and flexibility; however, it also reduced the electrical conductivity owing to the effective decrease in the concentration of PEDOT crystals (Figure S4, Supporting Information). At a lower alginate‐to‐PEDOT:PSS ratio, the higher effective concentration of PEDOT crystals and improved intercalated PEDOT structure afforded higher electrical conductivity. Based on these observations, the alginate/PEDOT:PSS‐glycerol(2.5/2‐9) glycerogel electrode was considered optimal. It exhibited good mechanical properties, including a tensile strength of 1.61 ± 0.08 MPa, elastic modulus of 5.51 ± 0.47 MPa, and stretchability of 64% ± 10%. In addition, it exhibited excellent electrical conductivity of 6.5 ± 0.2 S cm^−1^.

It should be noted that the air‐equilibrated final glycerogel electrode (air‐dried and annealed) and electrolyte (air‐dried) retained small amounts of water (≈6% and 9% of their final weight, respectively) due to the hygroscopic nature of glycerol. The slightly higher amount of water in the glycerogel electrolyte compared to the electrode is due to the presence of high‐density salt ions in the former gel. Nevertheless, major portion of both the glycerogel electrode and electrolyte was composed of polymer and glycerol. The excellent mechanical properties of the glycerogel electrode and electrolyte are attributed to the presence of strong yet sacrificial supramolecular aggregates in the gel network, which are formed by facile, strong, and reversible hydrogen bonding between glycerol and alginate (Figure 1a). To confirm the microstructure of the gels, scanning electron microscopy (SEM) analysis was performed, which revealed the formation of a high‐density interconnected aggregated domain structure in both the electrode and electrolyte (Figure S5, Supporting Information). The relatively higher density and finer aggregates in the electrode compared to the electrolyte is attributed to the presence of PEDOT crystals in the former gel system.

The glycerogel electrode exhibited a slightly decreased mechanical performance compared to its electrolyte counterpart. This was caused by the presence of a PEDOT:PSS network in the glycerogel electrode. PEDOT:PSS can enhance crystallinity but it does not possess any hydroxyl groups that may form an extensive hydrogen‐bonding network with alginate/glycerol, resulting in a slight decrease in stretchability and strength (Figure 2a,b). To confirm these findings, we also fabricated a control glycerogel electrode composed of pure PEDOT:PSS and glycerol without alginate for analysis (Figure S6, Supporting Information). Due to the complete absence of alginate, the gel exhibited a highly brittle nature with limited stretchability (24.2% ± 0.1%) and strength (0.96 ± 0.01 MPa).

To design our sustainable AGSC, the optically transparent alginate/LiClO_4_‐glycerol(3/3‐9) glycerogel (Figure 2c) was used as an electrolyte and separator, whereas the optically opaque alginate/PEDOT:PSS‐glycerol(2.5/2‐9) glycerogel (Figure 2d) was used to form active electrodes and current collectors. The uniform appearance of these glycerogels indicated that the components inside each material did not exhibit macro‐phase separation.

The molecular distribution of glycerol and its unique properties enabled the resulting gels to tolerate extreme temperatures. This phenomenon was demonstrated by exposing them to low (−20 °C) and high temperatures (80 °C) for 24 h, and immediately stretching them at respective temperatures (Figure 2e,f).

### 2.4 Self‐Healing, Degradation, and Rebuilding Capabilities of Reconfigurable Glycerogels

**Figure 3:**
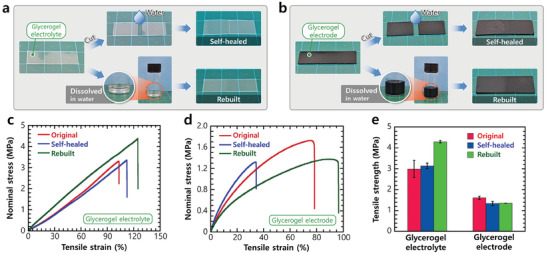
Self‐healing and rebuilding ability of the glycerogel electrolyte and electrode. Demonstration of the cutting/self‐healing and dissolution/rebuilding processes of the glycerogel a) electrolyte and b) electrode. Water acts as an environmentally friendly triggering agent that disrupts the supramolecular crosslinking structure of glycerogel, which is subsequently restored by air drying. Representative tensile stress–strain curves of the glycerogel c) electrolyte and d) electrode and e) their tensile strengths before and after self‐healing and rebuilding. Data in (e) are presented as the mean and mean absolute deviation (*n* = 3).

The water‐triggered self‐healing, degradation, and rebuilding capabilities of the glycerogel electrolyte and electrode are illustrated in **Figure** **3**a,b, respectively. When a small droplet of water was applied to the cut interface of the glycerogels, it was readily absorbed owing to the facile glycerol/water interactions. The absorbed water molecules interacted with the hydrogen‐bonding network, resulting in partial breakage of the polymer aggregates that enhanced the polymer dynamicity at the cut interfaces. Consequently, the cut surfaces coalesced via structural reconfiguration. Finally, the contacted gel specimen was air‐dried, allowing the glycerol molecules to regenerate the hydrogen‐bonding crosslinks within the polymer, thereby reconfiguring the internal structure to complete self‐healing.

The water‐triggered internal bonding tunability of our glycerogels also provided the capacity for structural degradation and rebuilding. Upon immersing the gels in a water bath, water molecules infiltrated the gel network. The glycerogels initially underwent swelling, followed by breakage, and eventually dissolved entirely in water by complete degradation of the aggregated bonding structures. The glycerogels could be restored to their original form by casting the degraded solution and air‐drying. Upon the completion of drying, the polymers and glycerol had rebuilt the original hydrogen‐bonded aggregate structure.

This water‐mediated self‐healing, degradation, and rebuilding approach was applicable to both the glycerogel electrolyte and electrode (Figure 3a,b). For the glycerogel electrolyte, the alginate hydrogen‐bonding structure was degraded upon the addition of water and rebuilt as it evaporated. Meanwhile, for the glycerogel electrode, both the alginate hydrogen‐bonding structure and the PEDOT:PSS crystalline structure were modulated by water. The overall dissolution speed of the glycerogel electrode was slower than that of the glycerogel electrolyte. For example, a glycerogel electrode (1 × 1 cm) required ≈6 h to completely dissolve in water, whereas a glycerogel electrolyte of similar size dissolved in ≈30 min.

We quantitatively assessed the self‐healing and rebuilding capacities of the reconfigurable glycerogels using tensile testing (Figure 3c–e). Both self‐healed and rebuilt glycerogel electrolytes exhibited similar or slightly higher tensile performance than the original electrolyte. Specifically, the tensile strengths of the healed and rebuilt glycerogel electrolytes were ≈105% and 142% of the original value, respectively (Figure 3e), and the elastic moduli were ≈99% and 189% of the original value, respectively (Figure S7, Supporting Information). The unexpected increase in these mechanical properties after rebuilding could be attributed to a slight reduction in the specimen thickness (≈80% of the original gel), likely due to the loss of some viscous precursor solution during transfer from the glass vial to the mold. This loss can be minimized when fabricating gels on a larger scale and such gels could exhibit a consistent performance after remodeling. The mechanical behavior of our thinner gel is consistent with the findings of our previous study,^[^ ^32^ ^]^ where we revealed that physically crosslinked polysaccharide gels tend to exhibit much improved mechanical behavior when their thickness is reduced. Conversely, the tensile strengths of the self‐healed and rebuilt glycerogel electrodes were slightly lower than that of the original electrode, at ≈82% and 83% of the original value, respectively (Figure 3e). The elastic moduli were ≈113% and 73% of the original value, respectively (Figure S7, Supporting Information). The slight reduction in self‐healing and rebuilding capacities for the glycerogel electrode, as compared to the glycerol electrolyte, were attributed to the crystalline PEDOT structure within the electrode, which is comparatively less sensitive to water. The presence of PEDOT crystals enabled a higher degree of crystallinity and aggregated structures in the glycerogel electrode compared to the electrolyte, and the SEM images (Figure S5, Supporting Information) show that denser and finer aggregates appeared in the former gel. The X‐ray diffraction profiles also demonstrated the presence of a higher degree of crystallinity in the glycerogel electrode compared to the electrolyte, as the former exhibited distinct crystalline peaks (2θ ≈ 21° and 26°)^[^ ^6^, ^14^ ^]^ with a lower full width at half maximum value (Figure S8, Supporting Information). Further, the inherent hydrophobic nature of PEDOT crystals and absence of hydroxyl groups on the PEDOT polymer makes the PEDOT:PSS aggregates less sensitive to water. These factors cause a slower dissolution of the electrode compared to the electrolyte. They also cause the slight reduction in the self‐healing and rebuilding capacities of the electrode compared to the electrolyte (Figure 3c–e). Nevertheless, the electrical conductivities of the self‐healed and rebuilt glycerogel electrodes were 6.5 ± 0.3 and 6.9 ± 0.4 S cm^−1^ (≈100% and ≈106% relative to that of the original gel), respectively (Figure S9, Supporting Information). This suggests the PEDOT percolation network was perfectly restored in the reconfigured glycerogel electrode.

**Figure 4:**
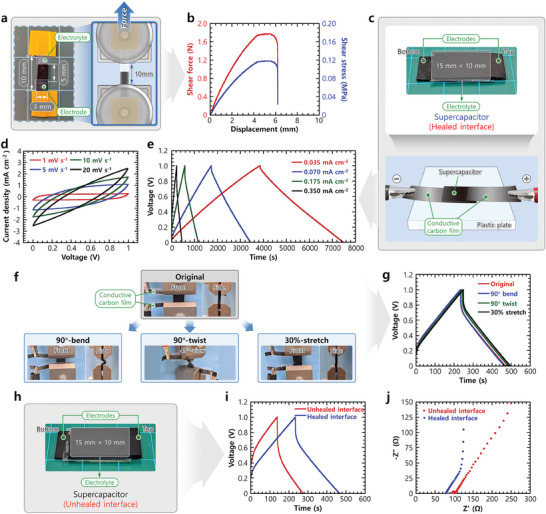
Interfacial and electrochemical characterization of the AGSCs. a) Photograph of the lap‐joint sample for analyzing the bonding strength of the self‐healed electrode/electrolyte interface and schematic of the lap‐shear test. b) Lap‐shear test results, demonstrating the shear adhesive force and strength of the self‐healed electrode/electrolyte interface. c) Photograph of a prototype AGSC with a healed electrode/electrolyte interface and schematic of the setup for electrochemical characterization. d) Cyclic voltammetry (CV) curves of AGSCs at different scan rates. e) Galvanostatic charge/discharge (GCD) curves of AGSCs at different current densities. f) Photographs of mechanically deformed AGSCs in different states (90° bend, 90° twist, and 30% stretch). g) GCD curves of an AGSC under different mechanical deformations at a current density of 0.350 mA cm^−2^. h) Photograph of an AGSC with an unhealed electrode/electrolyte interface. i) GCD curves at a current density of 0.350 mA cm^−2^ and j) Nyquist curves of AGSCs with unhealed and healed electrode/electrolyte interfaces.

Because the glycerogel electrode and electrolyte had similar structures, components, and compositions, they readily attached to one another via the water‐triggered reconfiguration mechanism. The electrode/electrolyte attachment efficiency was evaluated by fabricating a lap‐joint sample (**Figure** **4**a). Electrode and electrolyte strips (width: 3 mm) were placed in a longitudinal overlap configuration (overlap length: 5 mm). The interface between the samples was then healed by introducing a few droplets of water and allowing it to dry. Following this, the shear adhesive strength of the electrode/electrolyte interface was evaluated using a standard lap‐shear test (Figure 4b and Movie S3, Supporting Information). An excellent adhesive force of 1.82 ± 0.02 N was obtained, corresponding to an adhesive strength of 0.12 ± 0.01 MPa. Notably, the electrode/electrolyte lap‐joint sample could stretch by ≈60% of its initial length before experiencing failure, which closely matches the fracture strain of the individual components. This indicates that the toughness of the interface is similar to that of the bulk material. Thus, the excellent reconfiguration and self‐healing capacities of the glycerogel electrode and electrolyte facilitated the fabrication of a stretchable and reconfigurable AGSC.

### 2.5 Prototype AGSC Using Reconfigurable Glycerogels

#### 2.5.1 Electrochemical Performance

Figure 4c demonstrates a prototype AGSC with a sandwich structure (electrode/electrolyte/electrode) fabricated by a water‐triggered attachment method. This method involved attaching both surfaces of a glycerogel electrolyte (thickness: ≈0.45 mm) between two glycerogel electrodes (thickness: ≈0.8 mm). The active cross‐sectional area was 15 × 10 mm. Subsequently, CV and GCD tests were performed in a two‐electrode system to evaluate the electrochemical performance of our AGSC (Figure 4c–e). CV was performed at various scan rates within the voltage range of 0–1 V (Figure 4d). The CV curves exhibited a quasi‐rectangular shape at lower scan rates and lacked redox peaks, signifying that the charge‐storage mechanism was governed by electric double layer capacitance. The GCD curves at different current densities exhibited a symmetrical triangular shape within a potential window of 0–1 V, demonstrating the capacitive behavior of the supercapacitor (Figure 4e). The device exhibited an excellent areal capacitance of 126.51 mF cm^−2^ (mass‐specific capacitance: 9385.01 mF g^−1^) at a current density of 0.035 mA cm^−2^. Despite a ten‐fold increase in current density (0.35 mA cm^−2^), the device maintained a high areal capacitance of 63.66% (80.54 mF cm^−2^) (Figure S10, Supporting Information).

Thereafter, the AGSC was subjected to various complex mechanical deformations (e.g., bending, twisting, and stretching) to assess its electrochemical stability (Figure 4f). Even under extreme mechanical deformations, the device retained ≈100% of its original capacitance (Figure 4g). The CV and GCD curves in the deformed state were similar to those in the initial state, demonstrating the stable charge‐storage behavior of the AGSC under mechanical deformation (Figure 4g; Figure S11, Supporting Information). The exceptional performance of the supercapacitor during mechanical deformation results from the strong electrode/electrolyte interfacial connection and compatibility between the components in terms of mechanical properties. These factors ensure the structural integrity of the device, thereby enabling the applied force to be distributed uniformly across the polymer network and thus preventing interfacial failure.

The electrochemical properties of a supercapacitor with unhealed interfaces were also tested to emphasize the significance of interfacial healing (Figure 4h). In stark contrast to its healed counterpart, the unhealed device exhibited a reduction of 40.90% (47.6 mF cm^−2^) in its areal capacitance and a larger ohmic drop (*iR* drop) (≈40%) (Figure 4i). Further, while the Nyquist plot (Figure 4j) of the healed device demonstrated negligible charge‐transfer resistance at high frequencies and a short slope at mid‐frequencies, indicative of low electrolyte resistance,^[^ ^33^ ^]^ that of the unhealed device indicated higher internal resistance, resulting in substantial energy loss (Figure 4i). This is because the robustly healed electrode/electrolyte interface, facilitated by structural reconfiguration, provided a percolated pathway for ion diffusion. This reduced the internal resistance and enhanced the energy efficiency.

**Figure 5:**
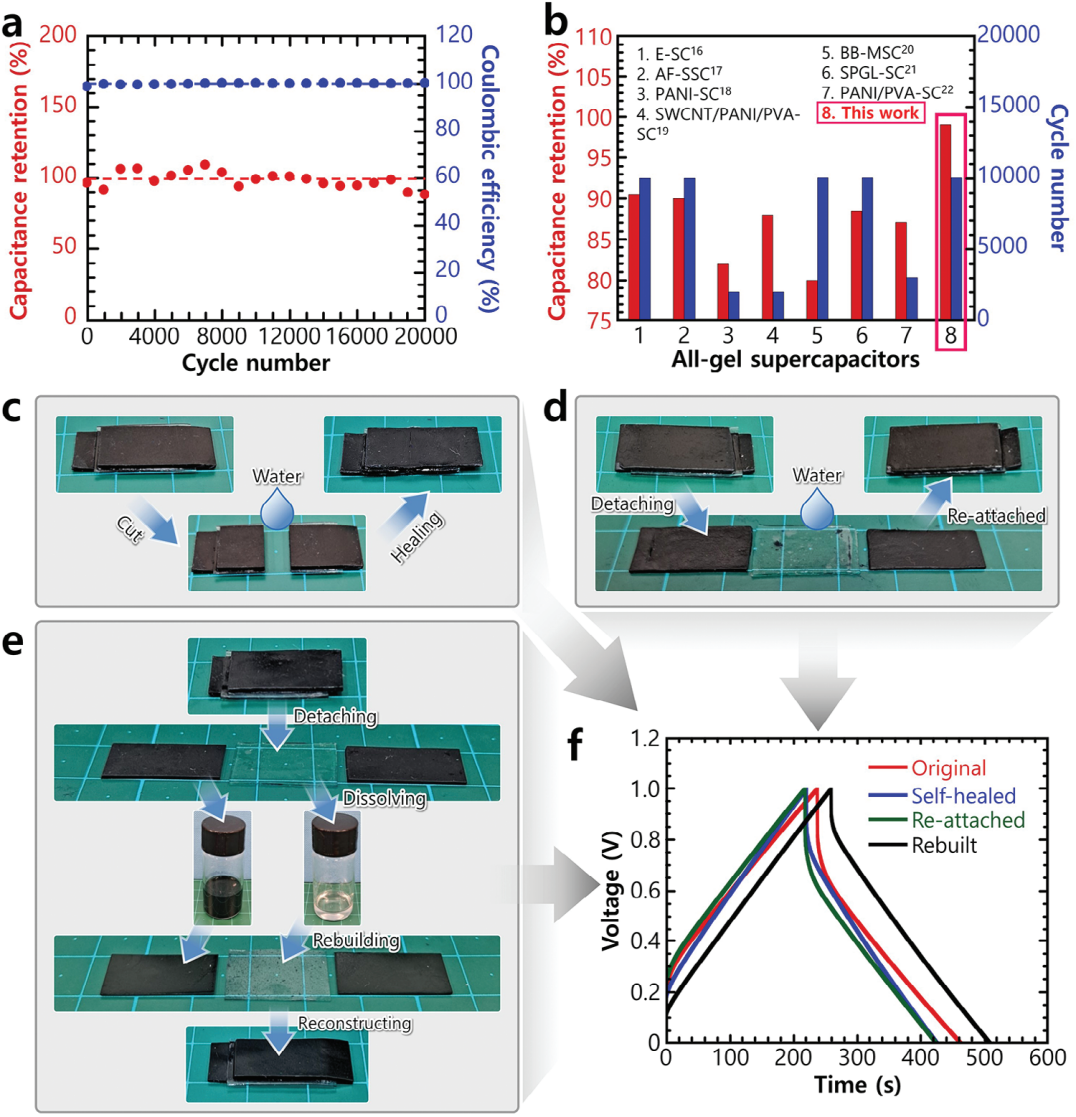
Electrochemical stability and reconfiguration ability of the AGSCs. a) Capacitance retention and Coulombic efficiency following 20 000 GCD cycles at a current density of 0.35 mA cm^−2^. b) Capacitance retention of the prepared AGSC and state‐of‐the‐art reported AGSCs. Demonstration of c) cutting/self‐healing, d) detaching/reattaching, and e) dissolution/rebuilding processes and f) GCD curves of the reconfigured AGSCs at a current density of 0.35 mA cm^−2^ demonstrating the electrochemical performance.

The electrochemical stability of the AGSC was characterized by subjecting it to 20 000 charge/discharge cycles at a current density of 0.35 mA cm^−2^ within the potential range of 0–1 V (**Figure** **5**a). After 10 000 and 20 000 cycles, the device exhibited capacitance retentions of ≈100% and 88.95%, respectively, along with an impressive Coulombic efficiency of >99.5%. This outstanding cycling stability is attributed to the well‐integrated internal structure of the glycerogel components, which comprise rigid polymers such as alginate and PEDOT:PSS. This structure forms a 3D hydrogen‐bonding network through facile polymer/solvent interactions. In addition, the AGSC has robust electrode/electrolyte interfaces. Consequently, its cycling performance surpasses that of several state‐of‐the‐art gel supercapacitors (Figure 5b and Table S1, Supporting Information).^[^ ^16^, ^17^, ^18^, ^19^, ^20^, ^21^, ^22^ ^]^

#### 2.5.2 Self‐Healing, Reattaching, and Rebuilding Capabilities

The sustainable usability of the designed AGSC was further demonstrated by its self‐healing, reattaching, and rebuilding (regeneration) capacities, which are derived from the water‐triggered switching ability of its physical bonds. This reconfiguration ability enabled the entire device to repair itself following cutting, simply by applying a few drops of water to the cut interface followed by air‐drying (Figure 5c). The reversible physical bonding at the electrode/electrolyte interface facilitated straightforward detachment when exposed to water and the reattachment of the components (Figure 5d). Furthermore, it enabled easy rebuilding (regeneration) of the device by detaching the components and dissolving them separately in water, regenerating them, and then reconstructing the device using the interfacial healing process (Figure 5e).

The reconfigured AGSCs maintained excellent electrochemical performance. The capacitances of the self‐healed and reattached devices were 91.01% and 87.50% of the original value, respectively (Figure 5f). Notably, the CV curves exhibited no apparent deviations from their original characteristics (Figure S12, Supporting Information). Despite undergoing molecular‐level rebuilding, the AGSC maintained an exceptional capacitance of 110.19% of the original value. The unexpected increase in capacitance, accompanied by a slight deviation in the CV characteristics post‐rebuilding, was attributed to the potential decrease in device thickness due to some loss of the precursor solution during the rebuilding process. The change in the thickness resulted in a slight variation of the charge‐storage kinetics of the rebuilt AGSC compared to the original AGSC, resulting in higher capacitance at a higher current density (0.35 mA cm^−2^). To adequately compare these systems, we also verified their capacitance at lower current densities (Figure S13, Supporting Information). The capacitance of the rebuilt AGSC at lower current densities (0.035−0.175 mA cm^−2^) was very close (91%–98%) to that of the original AGSC. Nevertheless, the reconfigured AGSC maintained high capacitance, indicating that the glycerogel electrode and electrolyte maintained their electrical and ionic conductivity after reconfiguration. This indicates that the device has the ability to seamlessly self‐heal, reattach, and reconstruct itself through molecular‐level reconfiguration via water‐triggering. Consequently, the AGSCs can be used sustainably for extended periods.

#### 2.5.3 Environmental Tolerance

**Figure 6:**
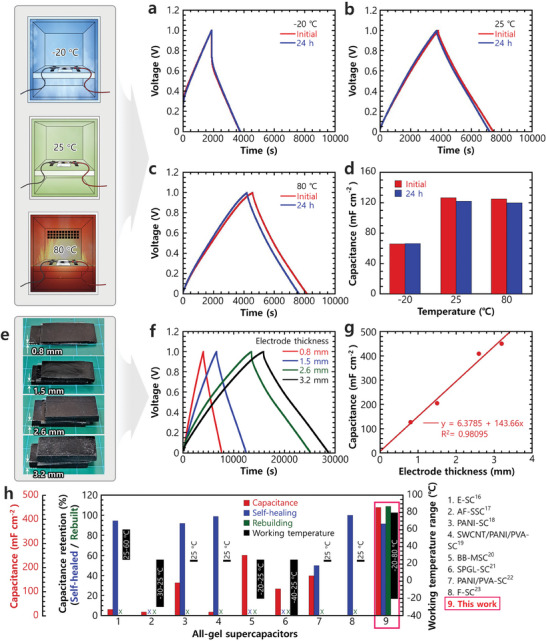
Electrochemical performance of AGSCs at extreme temperatures and their scalability. GCD curves at a current density of 0.035 mA cm^−2^ at a) −20, b) 25, and c) 80 °C, before and after 24 h incubation at the respective temperatures, and d) corresponding capacitance values. (e) Photographs of AGSCs fabricated from electrodes with varying thicknesses (0.8–3.2 mm) and f) their GCD curves at a current density of 0.035 mA cm^−2^. g) Relationship between capacitance (calculated from GCD curves) and electrode thickness. h) Performance of prepared and reported AGSCs in terms of capacitance, capacitance retention after self‐healing and rebuilding, and working temperature range.

An additional benefit of our AGSC is its ability to operate reliably across a wide temperature range. This benefit was assessed by conducting electrochemical tests on the device at different temperatures (−20, 25, and 80 °C) (**Figure** **6**a–d; Figure S14, Supporting Information). In contrast to conventional gel supercapacitors, which frequently experience operational issues at extremely low or high temperatures owing to solvent freezing or drying,^[^ ^12^, ^13^, ^14^, ^15^, ^16^, ^17^, ^18^, ^19^, ^20^, ^21^, ^22^, ^23^, ^29^ ^]^ our AGSC exhibited robust performance at temperatures as low as −20 °C and as high as 80 °C. Indeed, the GCD curves revealed impressive capacitance values of 65.73 and 125.26 mF cm^−2^ at −20 and 80 °C, respectively (Figure 6a,c). The extreme temperature tolerance of the AGSC was attributed to the inherent temperature stability of glycerol. In addition, the glycerol‐mediated hydrogen‐bonding network enables the polymer network to remain intact and function efficiently within a broad temperature range. The reduction in capacitance at low temperatures is caused by the enhanced viscosity of glycerol, which decreases the ionic conductivity.^[^ ^14^, ^29^ ^]^ Nevertheless, after incubating the AGSCs for 24 h at −20, 25, and 80 °C, the devices maintained excellent capacitances at 100.48%, 96.50%, and 95.64%, respectively, of the values prior to incubation (Figure 6a–d). Because the device was tested in an ambient environment, we also investigated its mass retention capabilities under extreme temperatures. After incubating the device at −20, 25, and 80 °C for 24 h, it maintained ±8 wt.% of its initial weight (Figure S15, Supporting Information), suggesting high‐level stability similar to our previously reported glycerogels.^[^ ^14^, ^29^ ^]^ A slight increase or decrease in weight could be due to the absorption of moisture from the air or a loss of the water retained in the gel. The aforementioned results indicate that the device remains operationally stable at extreme temperatures.

Next, we investigated the effect of humidity on device performance considering the hygroscopic nature of glycerol. When the device was maintained at a high humidity of 70%–80% for 24 h, a slight weight increase of ≈10% was observed due to moisture absorption. This moisture absorption led to a reduction in the equivalent series resistance in the higher frequency region of the Nyquist plot, indicating the increased ionic conductivity of the device (Figure S16a, Supporting Information). The absorbed moisture enhanced the ion mobility within the device, resulting in improved capacitance retention at higher current densities (Figure S16b, Supporting Information). Even after prolonged exposure to high humidity (70%–80%), the device maintained 100% of its areal capacitance at a current density of 0.035 mA cm^−2^, demonstrating a stable performance under humid conditions. However, when exposed to abnormally high humidity (>90%), the interface of the device weakened due to excessive moisture absorption, leading to the delamination of its components. However, the delaminated device can be reassembled by our water‐triggered self‐healing process (Figure 5d), which can provide stable performance in humid air (≤80%).

#### 2.5.4 Scalability and Generalizability

Another interesting feature of our AGSC is its scalability. Devices were fabricated with varying electrode thicknesses, and their electrochemical performance was evaluated (Figure 6e–g). For AGSCs with electrode thicknesses of 0.8, 1.5, 2.6, and 3.2 mm, the areal capacitances of the corresponding devices (measured through GCD) were 126.51, 204.49, 408.27, and 449.85 mF cm^−2^, respectively. This increase in areal capacitance with electrode thickness was attributed to the increased amount of active material in the thicker electrodes.

Conventional supercapacitors exhibit a negligible or minimal increase in capacitance with an increase in electrode thickness.^[^ ^34^ ^]^ In contrast, our AGSC displayed a sharp and linear increase in capacitance with increasing electrode thickness (Figure 6g) up to an electrode thickness of 3.2 mm; therefore, this thickness range (0.8–3.2 mm) can be considered as optimal for the electrode. This thickness range is well within that of commonly used electrodes for AGSCs. The optimal thickness may be selected depending on the energy requirements and packing limitations of specific applications. The 3D crosslinked alginate structure facilitated the incorporation of active materials, forming a percolated network even when the thickness was increased. Notably, the 3D structure enables facile ion diffusion, efficient charge storage at the molecular level, and high electrical conductivity. Therefore, our AGSC retains high electrochemical efficiency even at high thicknesses. The facile fabrication process, along with its scalability, demonstrates the adaptability of the AGSC for industrial applications that demand high energy densities. The excellent multifunctional performance of the AGSC significantly surpasses that of many state‐of‐the‐art gel supercapacitors, as evident from a comparison of their capacitance, capacitance retention after self‐healing and rebuilding, and working temperature range (Figure 6h).^[^ ^16^, ^17^, ^18^, ^19^, ^20^, ^21^, ^22^, ^23^ ^]^

In this study, we used an alginate‐based system to establish the fundamental strategy and fabrication process for water‐triggered reconfigurable glycerogels with tailored functionalities. However, the resulting gels exhibited limited stretchability (≤100%) because of the inherent rigidity of alginate. This limitation could restrict the range of potential applications. The selection of polymers and solvents is crucial in developing reconfigurable gels for diverse applications. The choice of polymer is limited to those that possess adequate physical crosslinking sites. Equally important is the choice of solvent, as different solvents exhibit varying crosslinking capabilities and might require different polymer densities to reach the gel point. This would ultimately affect the mechanical properties of the designed gels. Future studies should identify suitable polymers with a flexible backbone and abundant reconfigurable crosslinking capabilities. These polymers would offer enhanced stretchability and elasticity, making them suitable for use in various stretchable and wearable devices.

## 3 Conclusion

We devised a facile, bioinspired strategy to develop reconfigurable extremotolerant gels for sustainable application in next‐generation flexible and wearable devices. The approach involves a bottom‐up structure‐building process via the air‐drying of an aqueous biopolymer/glycerol‐based system. This results in the formation of biopolymer gels with a glycerol‐mediated supramolecular crosslinking structure. Similar to the structural remodeling process of biological tissues, these gels exhibit on‐demand structural switchability simply by water‐triggering owing to the facile mass exchange between the gel and sol phases. Furthermore, the inherent ability of glycerol to tolerate extreme temperatures and its molecular‐level involvement in forming a 3D crosslinked structure enables the resulting glycerogel to function effectively across a wide temperature range. This method can be scaled up and adapted to create various reconfigurable composite gels with diverse functions and applications.

We successfully utilized our bioinspired strategy to fabricate a reconfigurable glycerogel electrode and electrolyte for a high‐performance, sustainable AGSC. This supercapacitor possesses numerous functions, including self‐healing capacity, degradability, reconfigurability, wide temperature stability (−20 to 80 °C), and tolerance to mechanical deformation. Furthermore, the intercalated polymeric structure and robust mechanical and interfacial integrity result in remarkable capacitance (≈450 mF cm^−2^ at a current density of 0.035 mA cm^−2^) and impressive capacitance retention (≈89%) after 20 000 charge/discharge cycles (at a current density of 0.35 mA cm^−2^). Moreover, the device preserved ≈100% of its capacitance when subjected to extreme mechanical deformations such as bending, twisting, and stretching. Importantly, the device performed well at high and low temperatures of −20 and 80 °C, with minimal changes in capacitance (100% and 96% of the initial values, respectively) after incubation at these temperatures for 24 h. This extreme temperature tolerance was ascribed to the robust structure and inherent resilience of glycerol, which ensures reliable performance across a broad temperature range. The molecular‐level structural reconfigurability of the device ensured that it maintained excellent capacitance (91% and 110% of the original value at a current density of 0.35 mA cm^−2^) after self‐healing and rebuilding, respectively. This study provides a basis for the development of recyclable, self‐healing, and long‐lasting wearable and flexible energy storage devices with superior performance and sustainable usability.

## 4 Experimental Section

### Fabrication of Glycerogel Electrode and Electrolyte

The glycerogel electrode and electrolyte were fabricated independently using their respective precursor solutions. A typical precursor solution for the glycerogel electrode was prepared by dissolving 2.5 wt.% Na‐alginate, 2 wt.% PEDOT:PSS stock solution (2.5 wt.%, aqueous), and 9 wt.% glycerol in water. The electrode fabricated from this composition was used consistently throughout the study unless specified otherwise. Similarly, the precursor solution used for fabricating the glycerogel electrolyte was prepared by dissolving 3 wt.% Na‐alginate, 3 wt.% LiClO_4_, and 9 wt.% glycerol in water. Each solution was prepared inside a closed glass vial under constant magnetic stirring at room temperature (≈25 °C). After complete dissolution, the respective solutions were cast into flat glass molds with depths of 6.5 and 4 mm for the electrode and electrolyte, respectively. The filled molds were air‐dried in ambient conditions (temperature: ≈25 °C; humidity: 30–60%) for ≈3 days to promote gelation via water evaporation and thus obtain the as‐prepared glycerogels. Subsequently, the as‐prepared glycerol electrode was subjected to a closed dry‐annealing process inside a 120 °C oven for 10 min to obtain the final glycerogel electrode (thickness: ≈0.8 mm). The as‐prepared glycerogel electrolyte (thickness: ≈0.45 mm) was used directly following air‐drying without any further treatment. To investigate the effect of electrode thickness, glycerogel electrodes with various thicknesses (≈1.5, ≈2.6, and ≈3.2 mm) were fabricated by increasing the thickness of the cast solution (≈10, ≈15, and ≈20 mm, respectively). For comparison, a pure PEDOT:PSS glycerogel electrode was prepared by air‐drying a precursor solution containing 2 wt.% of PEDOT:PSS and 4 wt.% glycerol in water.

### Fabrication of Self‐Healed and Rebuilt Glycerogel Electrode and Electrolyte

To evaluate the self‐healing and rebuilding efficiencies of the glycerogel electrode and electrolyte, self‐healed and rebuilt gels were prepared from their pristine samples using the water‐triggered reconfiguration process.

For self‐healing, the sheet‐like glycerogel electrode (thickness: ≈0.8 mm) and electrolyte (thickness: ≈0.45 mm) were horizontally bisected along the center of their width using a sharp blade. The cut surfaces were moistened with a few drops of warm water (>60 °C) for ≈10 s and then rejoined in the original position. Because the use of excessive water can cause the deformation of bulk gel, only ≈30 µL of water per cm^2^ of the gel surface was used during the self‐healing process. The rejoined samples were air‐dried in ambient conditions (temperature: ≈25 °C; humidity: 30–60%) for 1 day to facilitate self‐healing via water evaporation. The resultant self‐healed glycerogel electrode and electrolyte were used for characterization.

For rebuilding, the sheet‐like glycerogel electrode (thickness: ≈0.8 mm) and electrolyte (thickness: ≈0.45 mm) were cut into small pieces and then completely dissolved in water (≈6 times the weight of the gel) while undergoing magnetic stirring. The obtained solution was subsequently utilized as the precursor solution to rebuild the glycerogel electrode and electrolyte using the fabrication procedure described previously. The final rebuilt glycerogels were characterized and compared with the original sample.

### Fabrication of AGSCs

The AGSCs were prepared by water‐triggered interfacial reconfiguration of glycerogel electrodes and electrolytes. First, the surfaces of the pre‐cut glycerogel electrode (length: 20 mm; width: 10 mm; and thickness: ≈0.8 mm) and electrolyte (length 20 mm; width: 10 mm; and thickness: ≈0.45 mm) were moistened by applying a few drops of warm water (>60 °C, ≈30 µL per cm^2^ of the gel surface). Subsequently, the glycerogel electrolyte was positioned between two glycerogel electrodes to form a sandwiched assembly with an overlapping area of 15 × 10 mm. This assembly was air‐dried in ambient conditions (temperature: ≈25 °C; humidity: 30–60%) for 1 day to facilitate self‐healing at the electrode/electrolyte interfaces through water evaporation. Finally, an assembled AGSC device with an active interfacial area of 15 × 10 mm was obtained. The extended parts (≈5 mm in length) of both electrodes were used to attach external connections during electrochemical measurements. AGSCs with thicker electrodes (≈1.5, ≈2.6, and ≈3.2 mm) were also prepared to investigate the effect of electrode thickness.

### Fabrication of Self‐Healed, Reattached, and Rebuilt AGSCs

AGSCs were reconfigured from their pristine states to demonstrate their reconfiguration ability. For self‐healing, the pristine AGSC was cut horizontally along the center of its width using a sharp blade. The cut surfaces were moistened with a few drops of warm water (>60 °C, ≈30 µL per cm^2^ of the gel surface), left for 1 min, and then carefully rejoined in the original position. The rejoined AGSC was air‐dried under ambient conditions (temperature: ≈25 °C; humidity: 30–60%) for 1 day to facilitate the repair of the interfacial structure through water evaporation, resulting in the formation of the self‐healed AGSC.

For reattachment, the pristine AGSC was exposed to a highly humid environment (>90%) for ≈30 min. This compromised the AGSC structure, enabling the weakened electrode and electrolyte layers to be carefully detached. The reattached AGSC was prepared by air‐drying the detached electrode and electrolyte gel sheets and then reassembling them into the original AGSC structure, following the previously described preparation process.

For rebuilding, the detached electrode and electrolyte gel sheets were cut into small pieces and then dissolved separately in water (≈6 times the weight of the gel) under magnetic stirring. Thereafter, the obtained electrode and electrolyte solutions were used to rebuild the respective gels, following the fabrication process described earlier. Subsequently, similar to the fabrication process of the original AGSC, the rebuilt glycerogel electrodes and electrolyte were assembled into a rebuilt AGSC.

Finally, the self‐healed, reattached, and rebuilt AGSCs were characterized, and the data were compared to those of the original AGSC.

## Conflict of Interest

The authors declare no conflicts of interest.

## Supporting information



Supporting Information

Supplemental Movie 1

Supplemental Movie 2

Supplemental Movie 3

## Data Availability

The data that support the findings of this study are available in the supplementary material of this article.
