# Supplementary material for: Water‐Triggered Reconfigurable Glycerogels for Sustainable All‐Gel Supercapacitors
Source: Adv Sci (Weinh). 2024 Dec 4;12(4):2411847. doi: 10.1002/advs.202411847 (PMC11775550; doi:10.1002/advs.202411847)
Supplement: Supplementary file 1 — Supporting Information [file ADVS-12-2411847-s002.docx]

Supporting Information

Water-Triggered Reconfigurable Glycerogels for Sustainable All-Gel Supercapacitors

Md. Tariful Islam Mredha, Adith Varma Rama Varma, Tanish Gupta, and Insu Jeon*

**Materials and characterization**

*Materials:* Sodium alginate (Na-alginate), poly(3,4-ethylenedioxythiophene)-poly(styrenesulfonate) (PEDOT:PSS; dry re-dispersible pellets), lithium perchlorate anhydrous (99%), glycerol (99%), and carbon nanotube films were obtained from Junsei Chemical Co., Ltd. (Japan), Sigma-Aldrich (Belgium), Thermo Scientific (USA), Samchun Pure Chemicals and Metals (Republic of Korea), and Chengdu Organic Chemicals (Chinese Academy of Sciences, China), respectively. Ultrapure deionized water was used for all experiments as required.

*Mechanical Characterizations:* A universal testing machine (TEST ONE TO-100-1C, Republic of Korea) equipped with a 10-kgf load cell was used to perform all mechanical characterizations. All tests were conducted under ambient conditions (humidity: 30%–60%; temperature: ~25 °C).

For tensile testing, the samples were cut into rectangles (length: ~25 mm; width: ~3 mm) and mounted into the tensile tester by clamping both ends along the longitudinal direction, ensuring a distance of ~10 mm between the clamps. The tests were conducted by moving the upper clamp upward at a constant deformation rate of 500% min^−1^, unless specified otherwise. To evaluate the healing and rebuilding efficiencies, tensile tests were conducted on self-healed and rebuilt samples using similar test parameters (e.g., sample size and deformation speed). Each gel type was tested in triplicate, and the average values were obtained.

Lap shear tests were conducted to evaluate the bonding strength of the self-healed interface between the glycerogel electrolyte and electrode. The surfaces of the glycerogel electrolyte strip (width: ~3 mm; thickness: ~0.45 mm) and glycerogel electrode strip (width: ~3 mm, thickness: ~ 0.8 mm) were moistened with warm water (>60 °C, ~30 μL per cm^2^ of the gel surface) and stacked with a longitudinal overlap of ~5 mm. This assembly was subsequently left in ambient conditions (temperature: ~25 °C, humidity: 30%–60%) for 1 d for interfacial self-healing through water evaporation. To perform the lap shear tests, the ends of the lap-joint sample were mounted into the clamps of the tensile tester, ensuring a ~10 mm initial distance between the clamps. The tests were conducted at a constant deformation rate of 500% min^−1^.

*X-ray diffraction (XRD):* The crystallinity of the glycerogel electrode and electrolyte was evaluated using XRD measurements (PANalytical EMPYREAN) with a CuKα-radiation source (0.1541874 nm) at a diffraction angle (2θ) of 5°−40°.

*Scanning electron microscopy (SEM):* The morphology of glycerol electrode and electrolyte was characterized using field-emission SEM (S-4700, Hitachi, Japan). The solvent in the glycerogel samples was sequentially exchanged with ethanol. The samples were then transferred into a liquid nitrogen bath, where the cross-sectional surfaces were exposed by breaking the samples under frozen condition. Then samples were freeze-dried for 24 h. The freeze-dried samples were coated with platinum using an ion-sputtering device and their morphology at cross-sectional surfaces was observed through SEM.

*Weight stability:* Weight changes of the AGSC at different temperatures (-20, 25, and 80 °C) and humidities were monitored over a period of 24 h. The weight fraction of the device was calculated from the ratio of the final weight (*W*_f_) to the initial weight (*W*_i_):

Weight fraction = $\frac{W_{f}}{W_{i}}$

*Water content measurement:* The water content in the final glycerogel electrode and electrolyte was determined via the difference between the initial weight (*W*_i_) and the weight of the completely dried gels after exposure to 100 °C for 24 h (*W*_d_).

Water content (wt%) = $\frac{W_{i}-W_{d}}{W_{i}} 100\%$

*Measurement of active material (PEDOT:PSS) in the glycerogel electrode:* The PEDOT:PSS loading (active mass) in glycerogel electrode was determined as follows. The initial weight (*W*_i_; g) of a glycerogel electrode sheet (area: 1 cm^2^, thickness: ~0.8 mm) was measured. The combined weight (g) of alginate, PEDOT:PSS and glycerol in that electrode sheet was calculated as 94% of *W*_i_ (by eliminating 6% part residual water from *W*_i_). This value was then substituted into the following equation:

Active mass (g) = $\frac{y}{x+y+z} {0.94W}_{i}$

where *x*, *y*, and *z* are the ratios of alginate, PEDOT:PSS, and glycerol used in the precursor solution, respectively.

The active mass loading of the glycerogel electrode (alginate/PEDOT:PSS-glycerol(2.5/2-9)) with a thickness of ~0.8 mm was calculated as 13.48 mg cm^-2^_._

*Electrical Conductivity Measurements:* Glycerogel electrode strips (length: ~25 mm; width: ~3 mm; thickness: ~0.8 mm) were used for electrical conductivity measurements. The resistance of each specimen was measured under ambient conditions (humidity: 30%–60%; temperature: ~25 °C) using a precision LCR meter (4284A, Hewlett-Packard, USA) connected to Kelvin probes. The electrical conductivity (*σ_elec_*; S cm^−1^) was calculated using the following equation:

${}_{elec}=\frac{l}{RA}$

where l, R, and A denote the length, resistance, and cross-sectional area of the gel specimen, respectively. Three samples were tested for each gel type and the results were averaged.

*Electrochemical Analyses:* All the electrochemical analysis was performed using multichannel potentiogalvanostat (Metrohm Multi Autolab/M204, Netherlands) and a battery cycler (WBCS3000; WonATech, Republic of Korea). All tests were conducted under ambient conditions (humidity: 30%–60%; temperature: ~25 °C), unless otherwise specified.

The ionic conductivity of the glycerogel electrolyte was measured using electrochemical impedance spectroscopy (EIS). A glycerogel electrolyte sheet (length × width × thickness: 1.5 mm × 1 mm × 0.45 mm) was placed between two stainless-steel plates and EIS was performed in the frequency range of 10^6^–0.1 Hz at an amplitude of 10 mV. The resistance (*R*) of the sample was determined from the X-axis intercept value at highest frequency region of the Nyquist plot. The ionic conductivity (*σ_ionic_*; S cm^−1^) was calculated using the following equation:

${}_{ionic}=\frac{l}{RA}$

where *l* and *A* denote the thickness and cross-sectional area of the gel specimen, respectively.

For the electrochemical characterizations of the developed all-gel supercapacitor (AGSC) carbon nanotube films were attached to the outer extended edges of the AGSC, which acted as a wire for connecting the measurement probes. Cyclic voltammetry and galvanostatic charge–discharge measurements were performed at scan rates of 1–100 mV s^−1^ and current densities of 0.035–0.350 mA cm^−2^, respectively, over a potential window of 0–1 V. EIS was performed in a frequency range of 10^6^–0.005 Hz at an amplitude of 10 mV. The areal capacitance (*C*_ac_) was calculated as:

$C_{\mathrm{ac}}=\frac{i \times\Delta t_{d}}{A \times\Delta V}$

where *i*, Δ*t*_d_, *A*, and ΔV are the current, discharge time, active surface area of the supercapacitor, and voltage window of the measurement, respectively.

The mass specific capacitance (*C*_sc_) was calculated using the following equation:

$C_{sc}=\frac{i\times t_{d}}{m\times\Delta V}$

where *i*, *t*_d_, *m*, and Δ*V* are the current, discharge time, total active mass of the supercapacitor, and voltage window of the system, respectively.

Electrochemical analysis was conducted on the pristine, self-healed, re-attached, and rebuilt AGSCs to evaluate their basic and reconfiguration performance. The performance of the AGSCs under various mechanical deformation conditions was evaluated by deforming them using a tensile machine and subsequently measuring their electrochemical performance at the given state of bending, twisting, or stretching. To assess the electrochemical stability of the AGSC at different temperatures and humidity, the device was maintained at the given temperature or humidity for 24 h, followed by data collection. AGSCs with varying electrode thicknesses were tested to evaluate their scalability.

**Supplementary Figures**


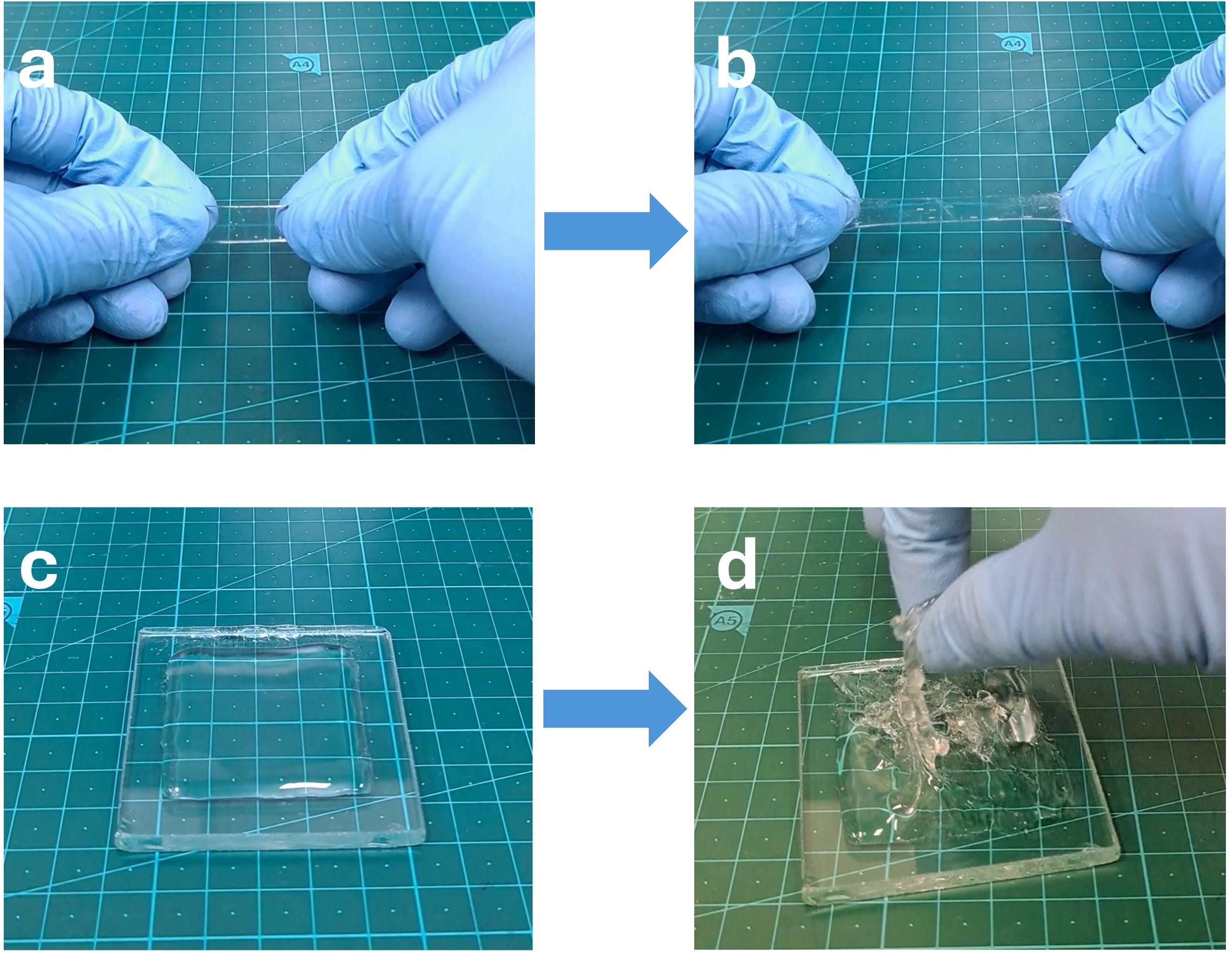


**Figure S1.** (a) A precursor solution containing 3 wt% alginate and 27 wt% glycerol in water was completely air-dried to obtain a reach a final composition of 10 wt% alginate in glycerol. (b) The air-dried sample was a stable 3D gel, as demonstrated by stretching it beyond 100%. (c) A precursor solution containing 3 wt% alginate in water was air-dried to a polymer concentration of 10 wt% in water. (d) The air-dried sample was a viscous slurry.


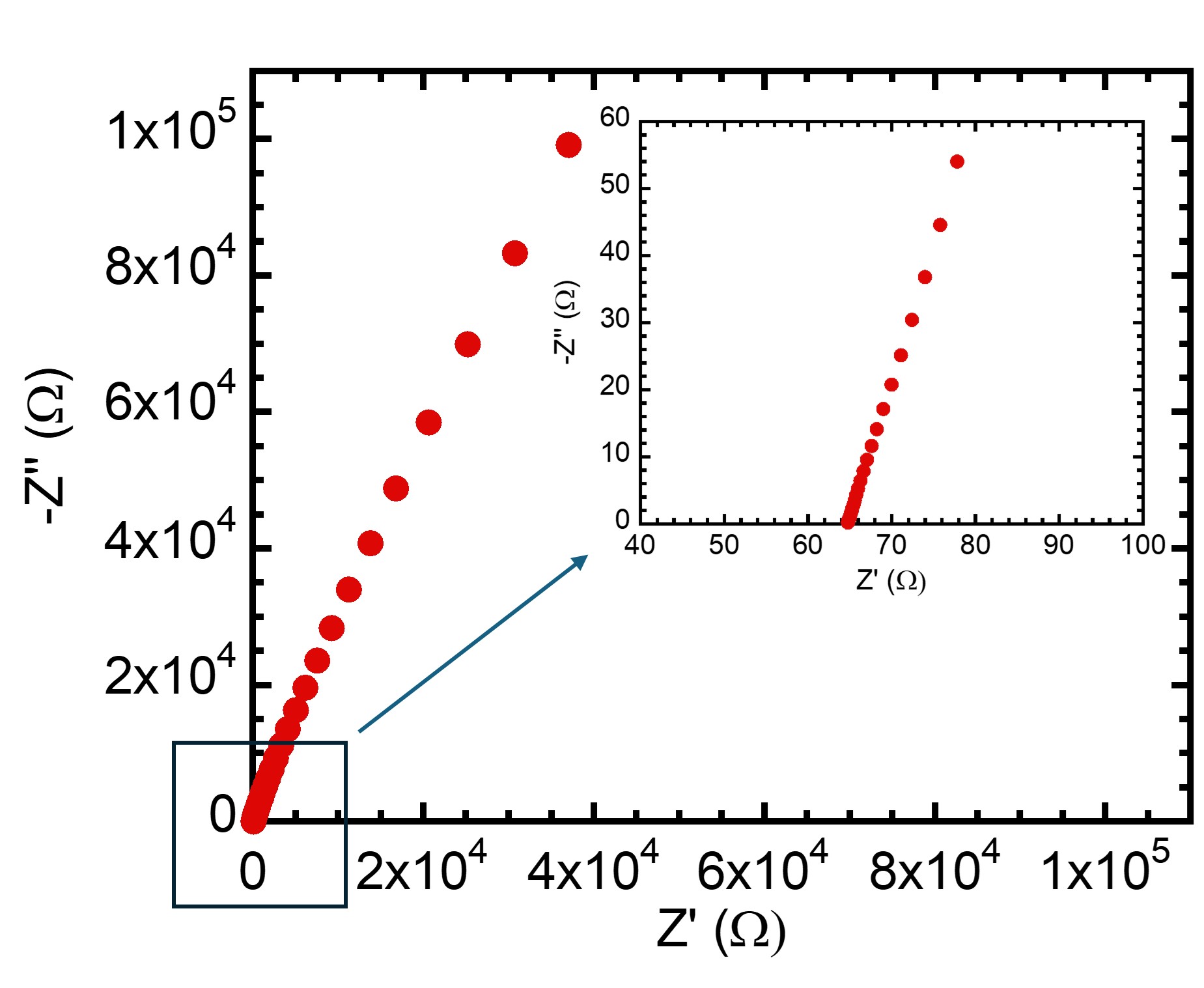


**Figure S2.** Nyquist plot of the glycerogel electrolyte. The magnified section, displayed as an inset image, represents the X-axis intercept in the higher frequency region of the Nyquist plot.


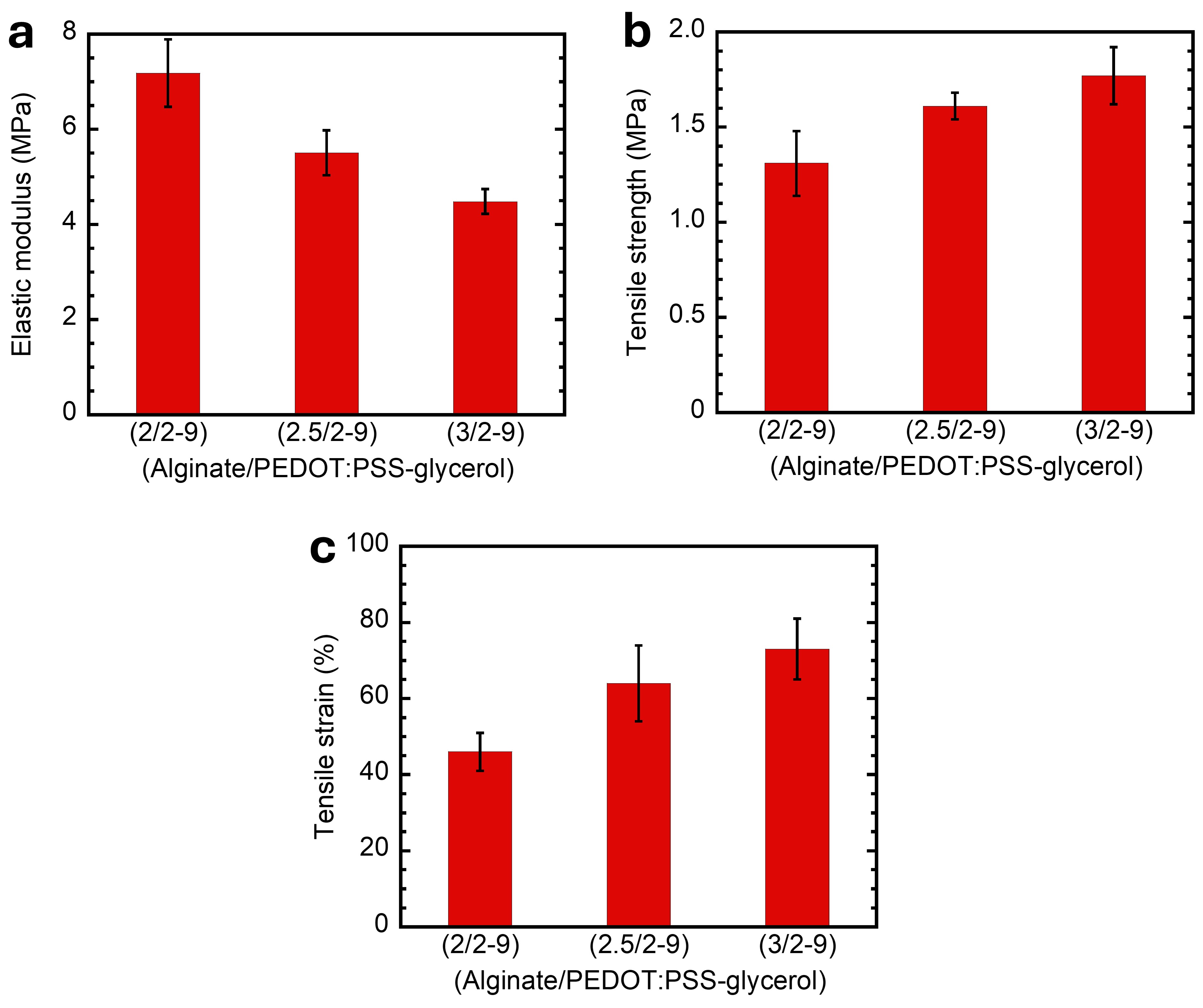


**Figure S3.** (a) Elastic modulus, (b) tensile strength, and (c) tensile strain of reconfigurable glycerogel electrodes with different alginate concentrations. Data are presented as mean with absolute deviations (*n* = 3).


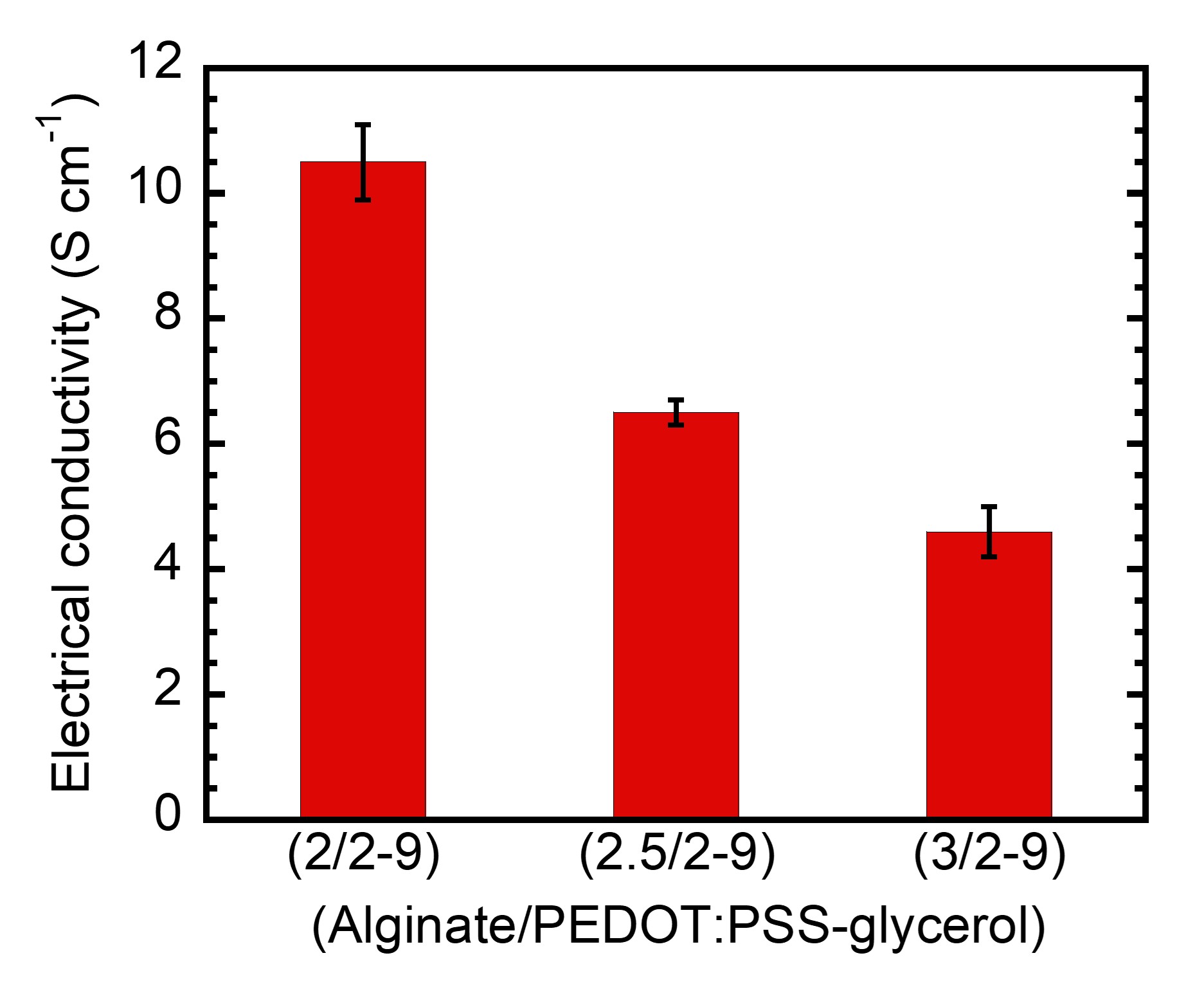


**Figure S4.** Electrical conductivity of reconfigurable glycerogel electrodes with different alginate concentrations. Data are presented as mean with absolute deviations (*n* = 3).


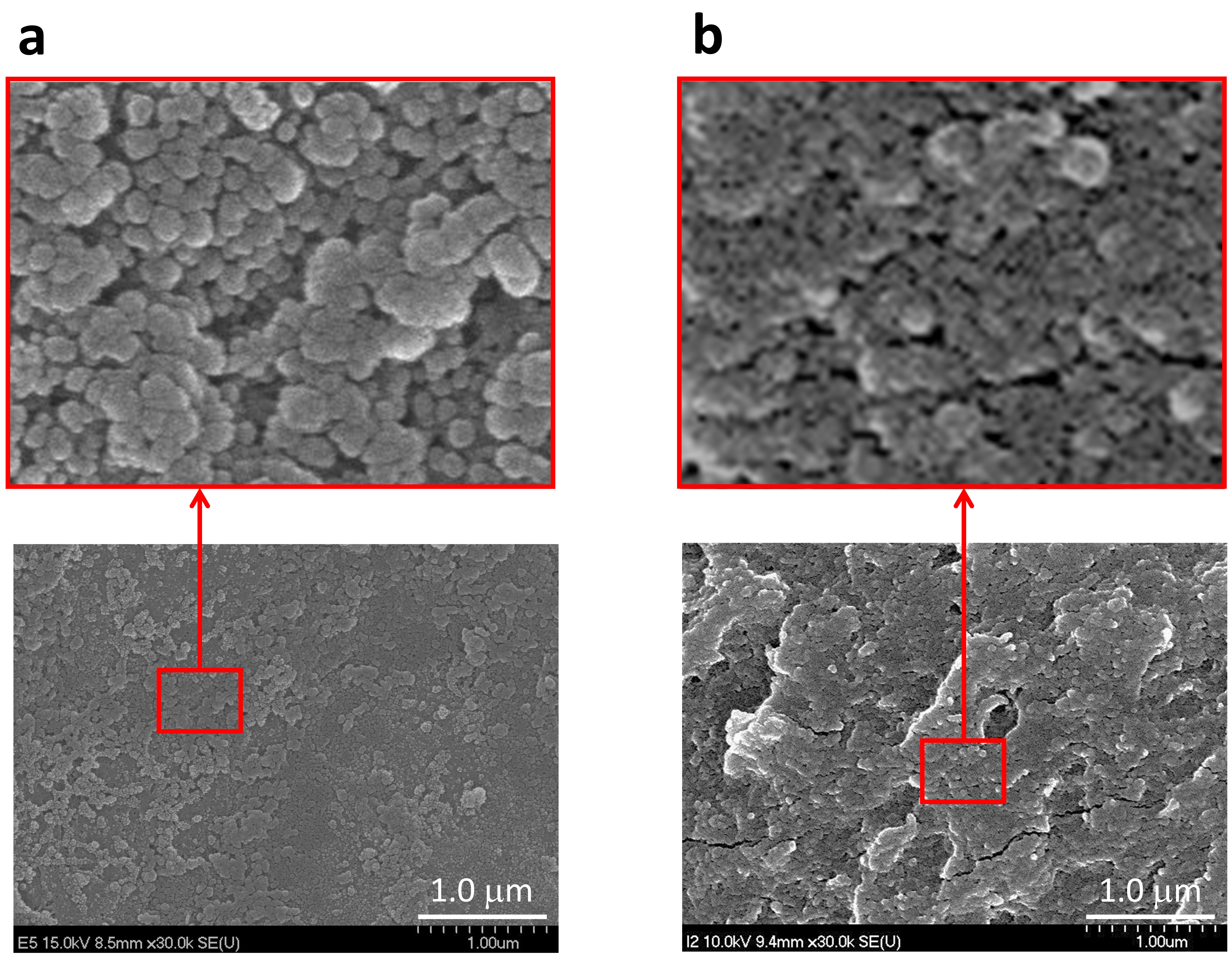


**Figure S5.** SEM images of the glycerogel (a) electrode and (b) electrolyte.

**
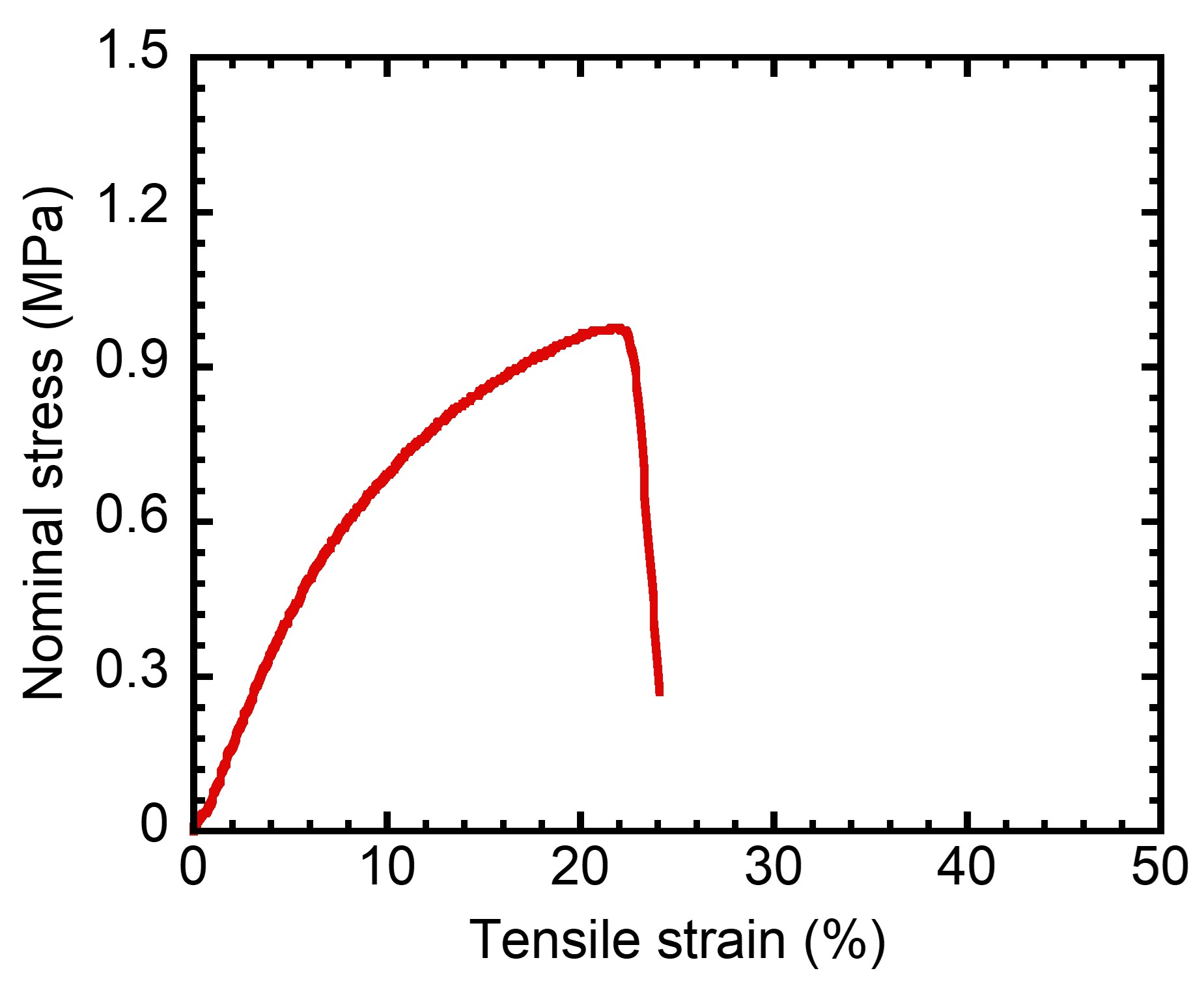
**

**Figure S6.** Representative tensile stress–strain curve of PEDOT:PSS-glycerol(2-4) glycerogel.


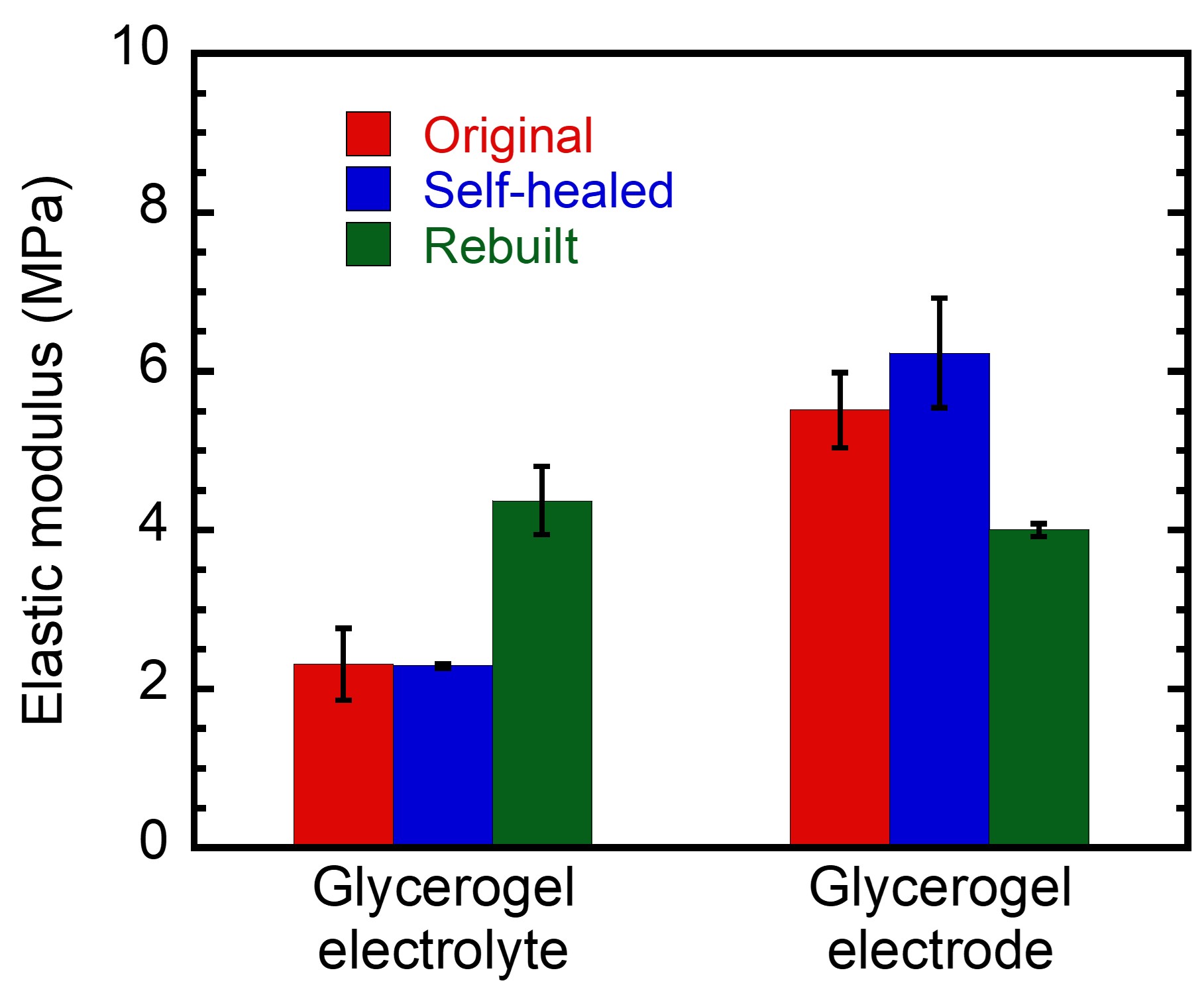


**Figure S7.** Elastic modulus of reconfigurable glycerogel electrolyte and electrode before and after self-healing and rebuilding. Data are presented as mean with absolute deviations (*n* = 3).


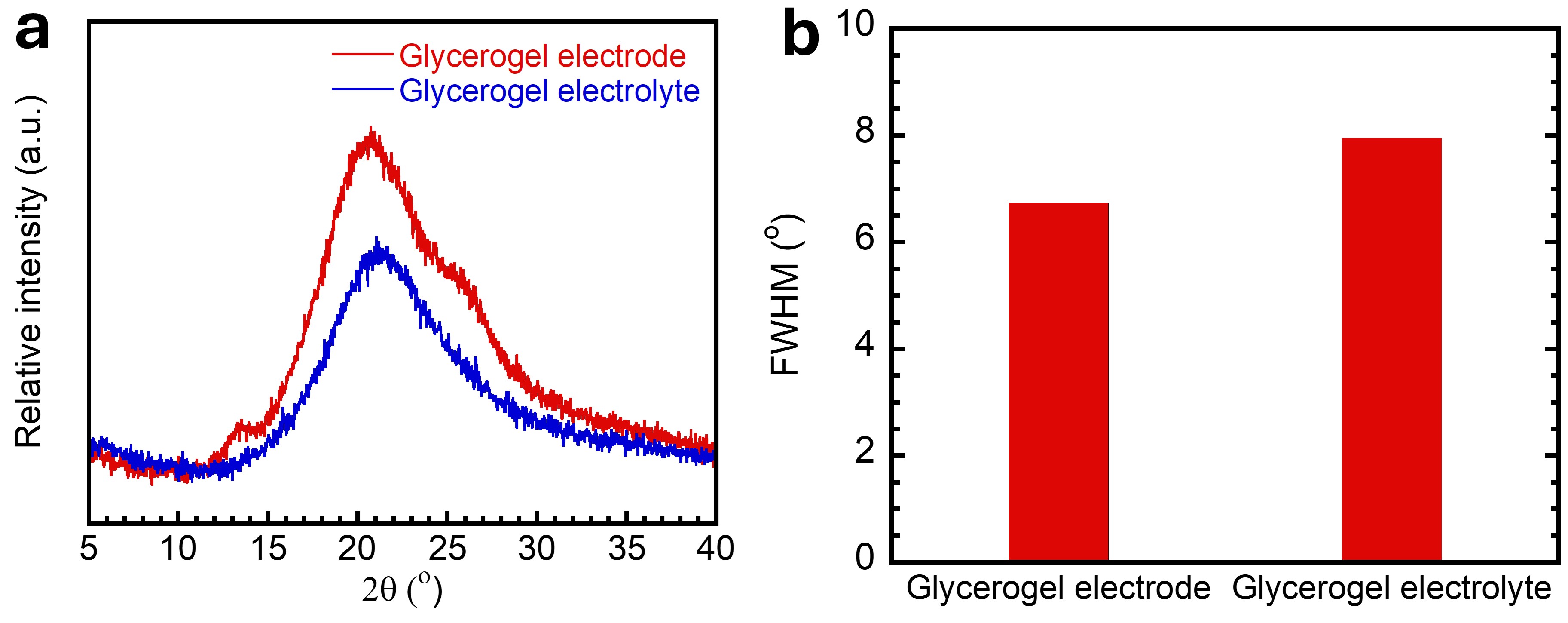


**Figure S8.** (a) XRD curves of the glycerogel electrode and electrolyte. (b) Full width at half maximum (FWHM) values of these gels for the peak at 2θ ≈ 21°.


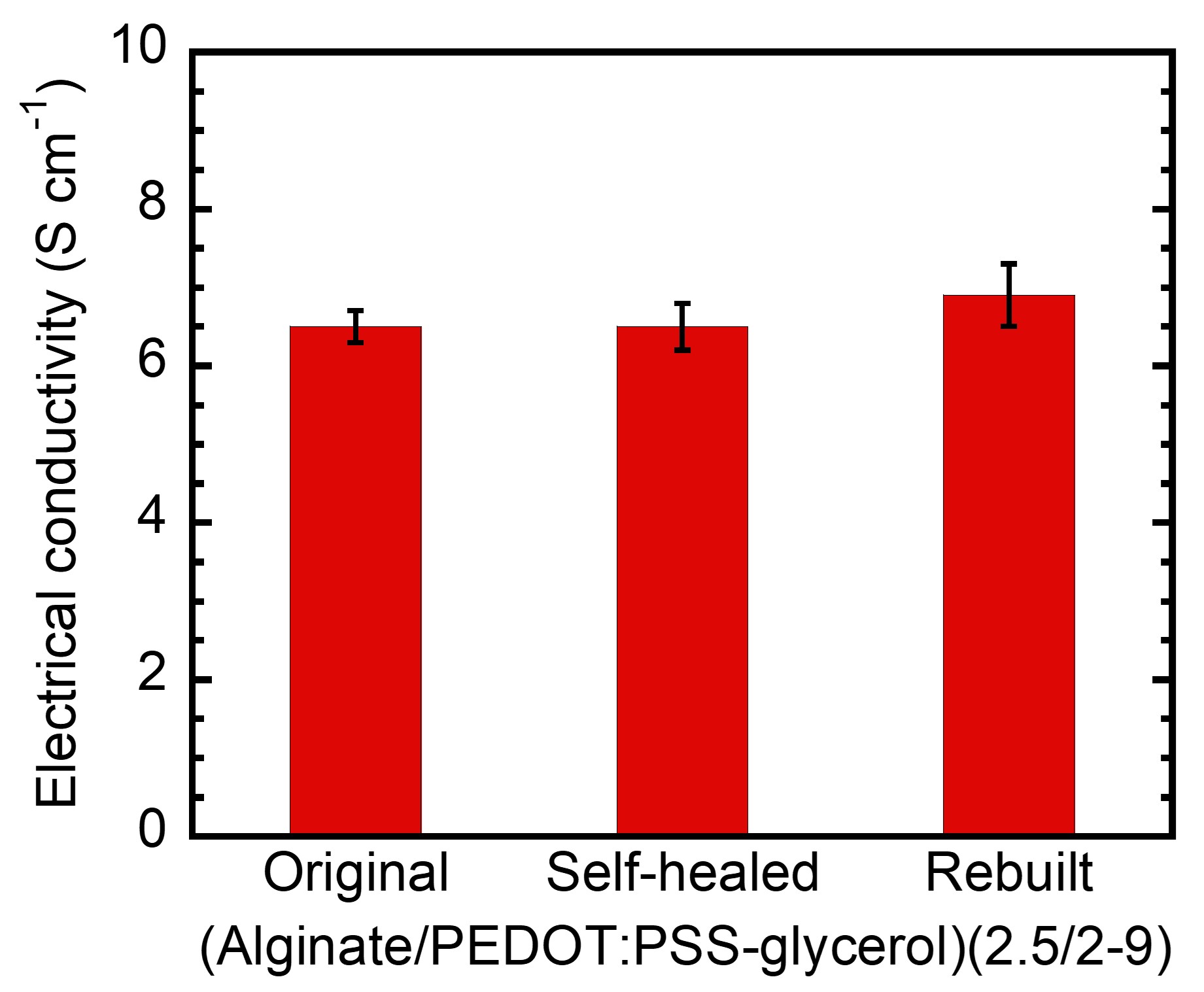


**Figure S9.** Electrical conductivity of reconfigurable glycerogel electrode before and after self-healing and rebuilding. Data are presented as mean with absolute deviations (*n* = 3).


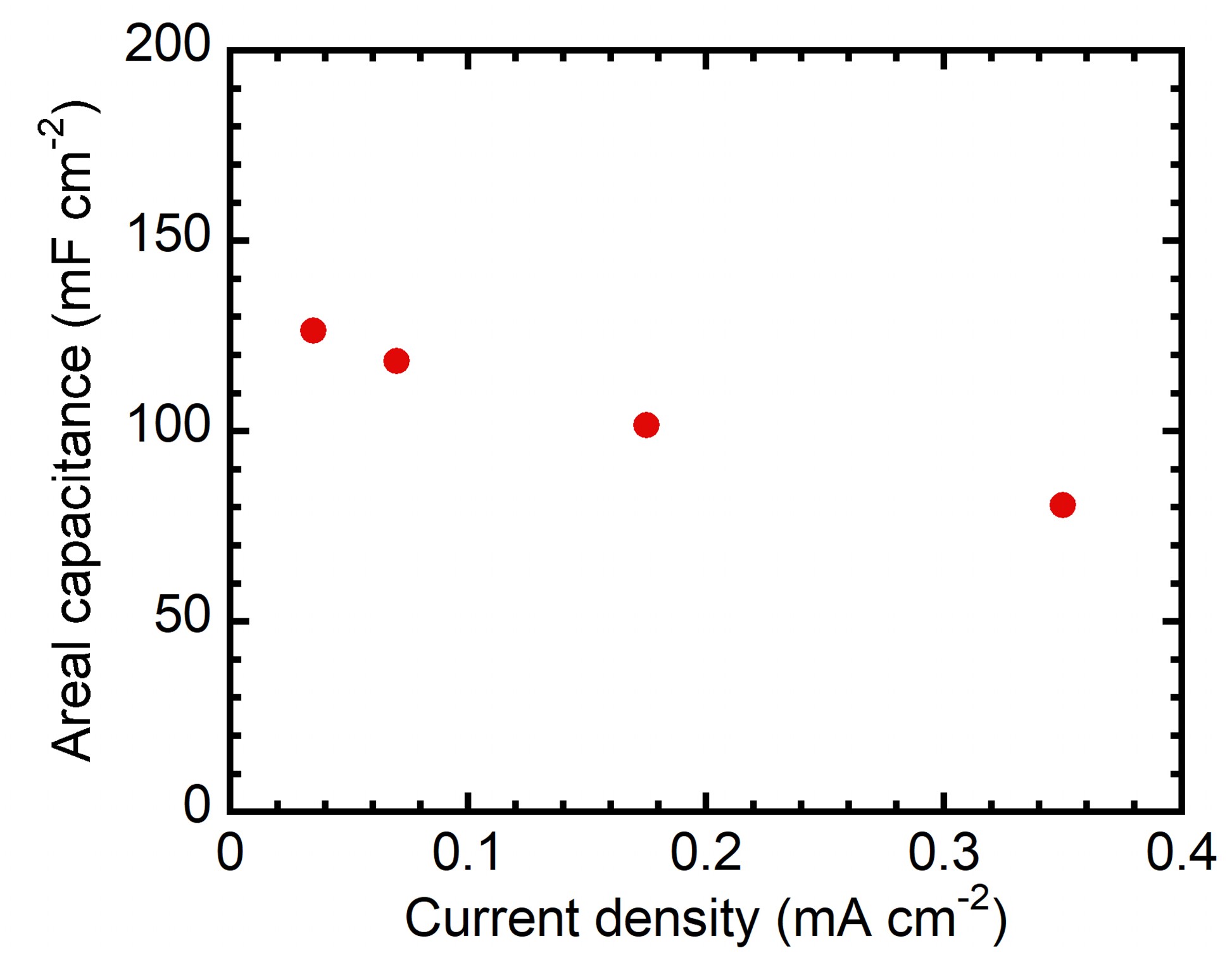


**Figure S10.** Areal capacitance of AGSCs as a function of current density.


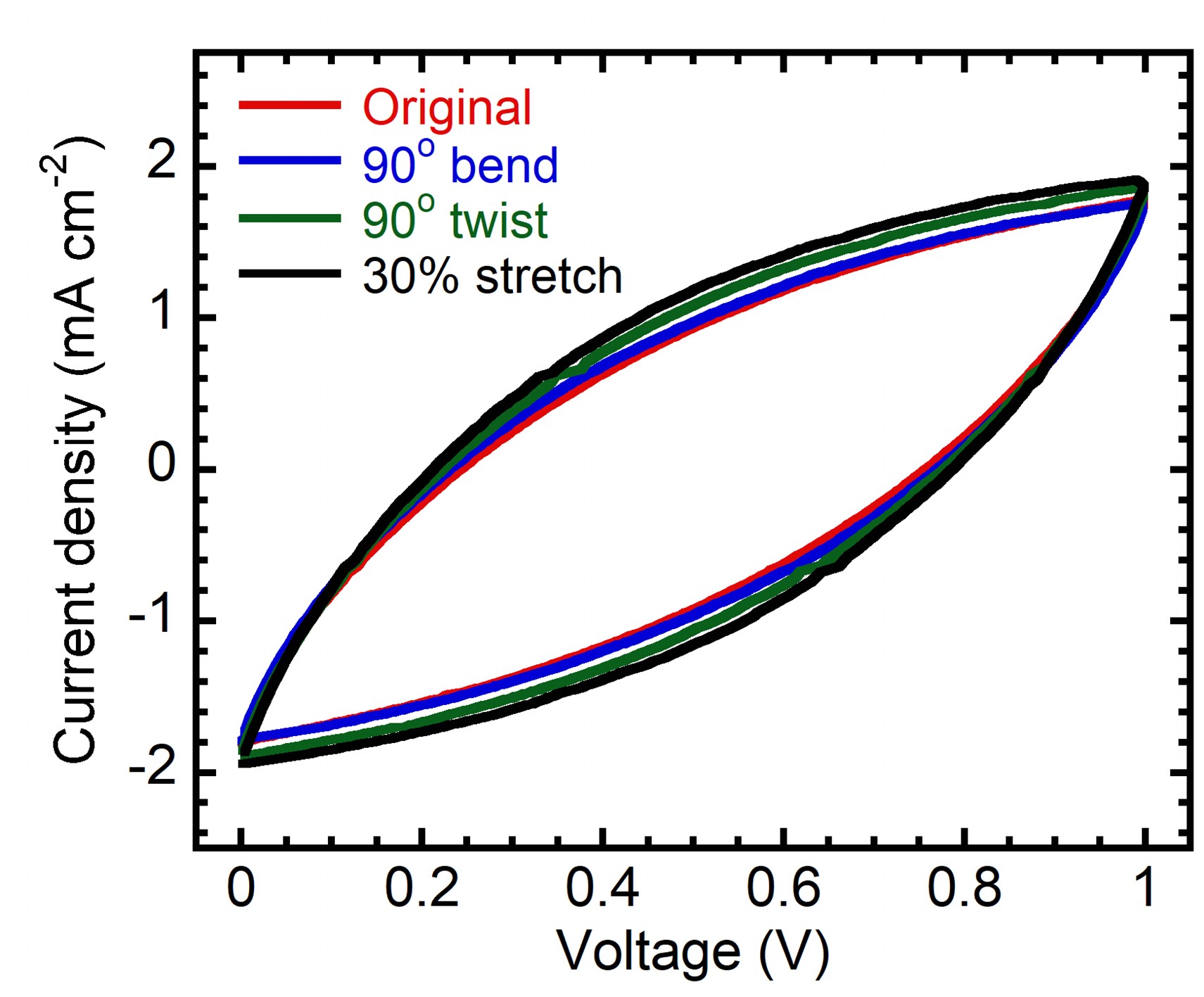


**Figure S11.** Cyclic voltammetry curves of AGSCs (scan rate: 10 mV s^−1^) under bending, twisting, stretching, or no deformation.


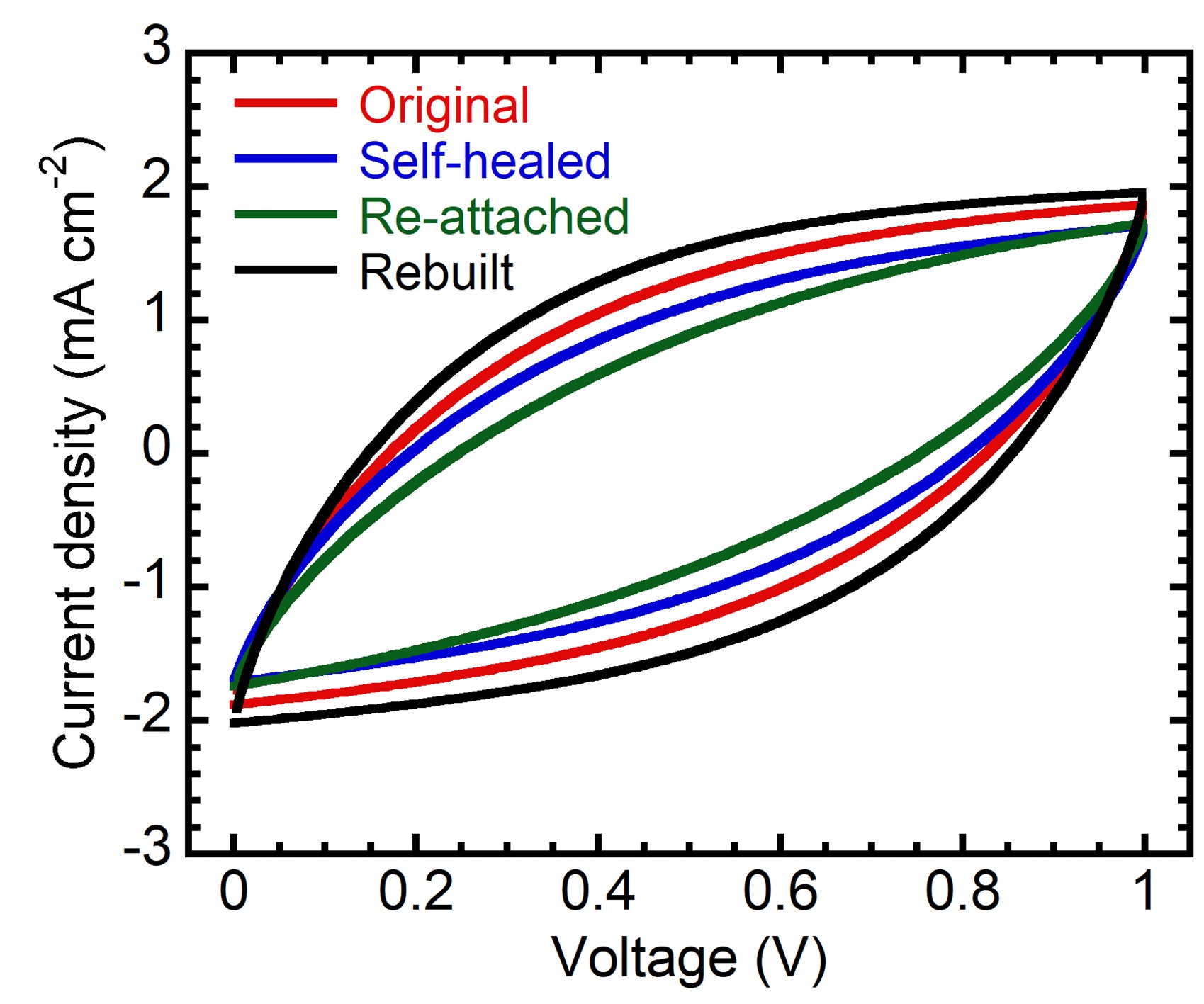


**Figure S12.** Cyclic voltammetry curves of AGSCs (scan rate: 10 mV s^−1^) before and after self-healing, re-attaching, and rebuilding.


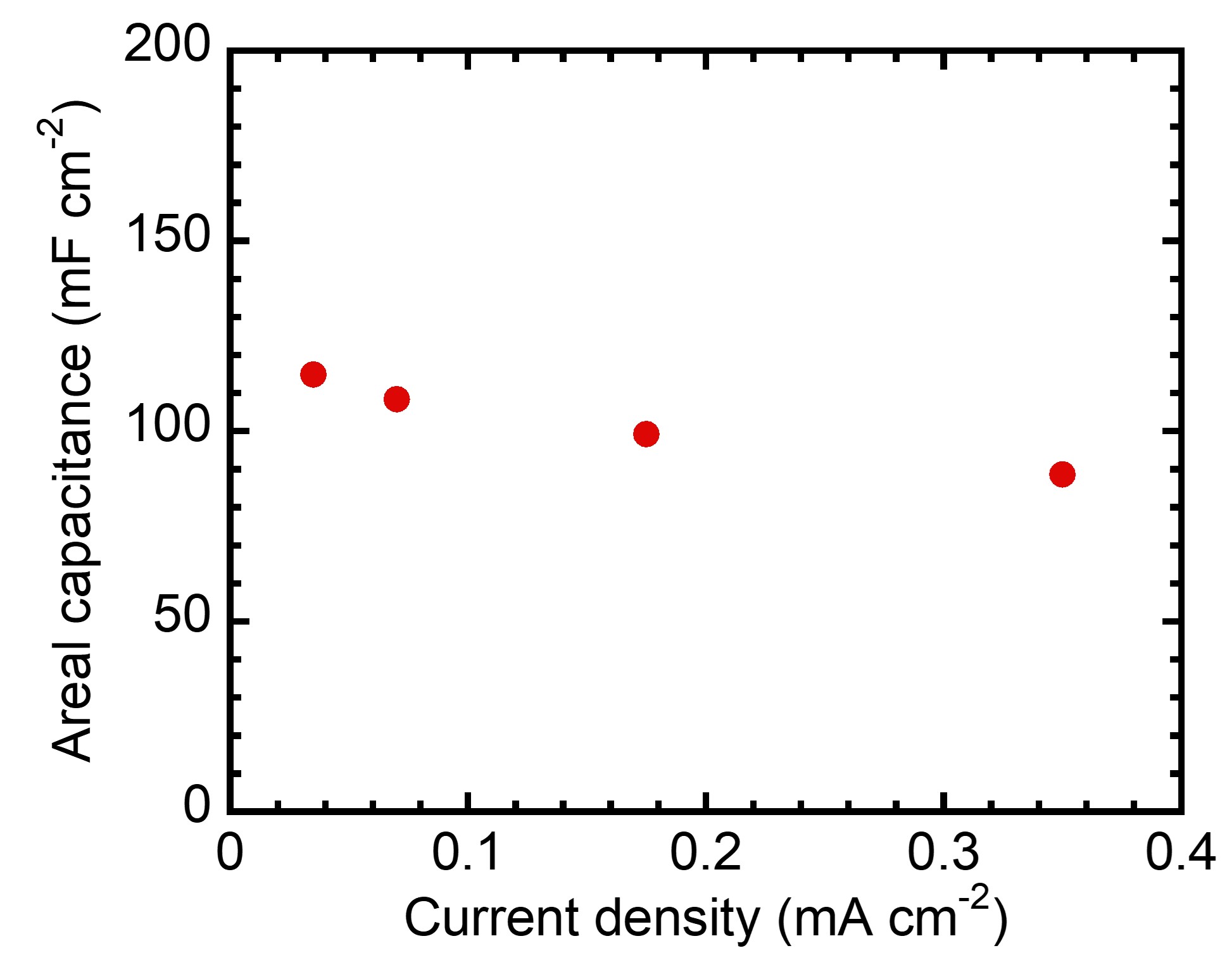


**Figure S13.** Areal capacitance of the rebuilt AGSC as a function of current density.


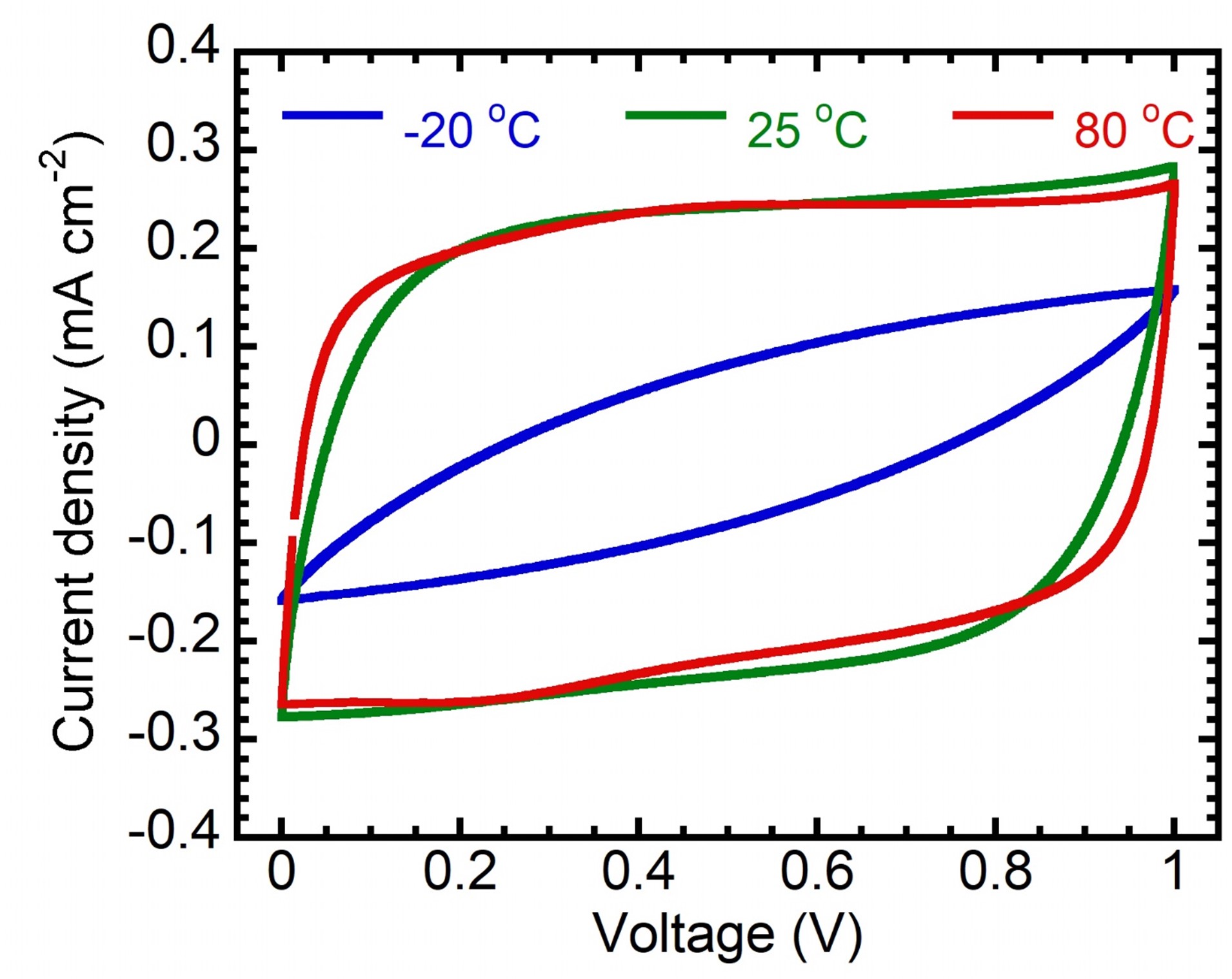


**Figure S14.** Cyclic voltammetry curves of AGSCs (scan rate: 10 mV s^−1^) at temperatures of −20, 25, and 80 °C.


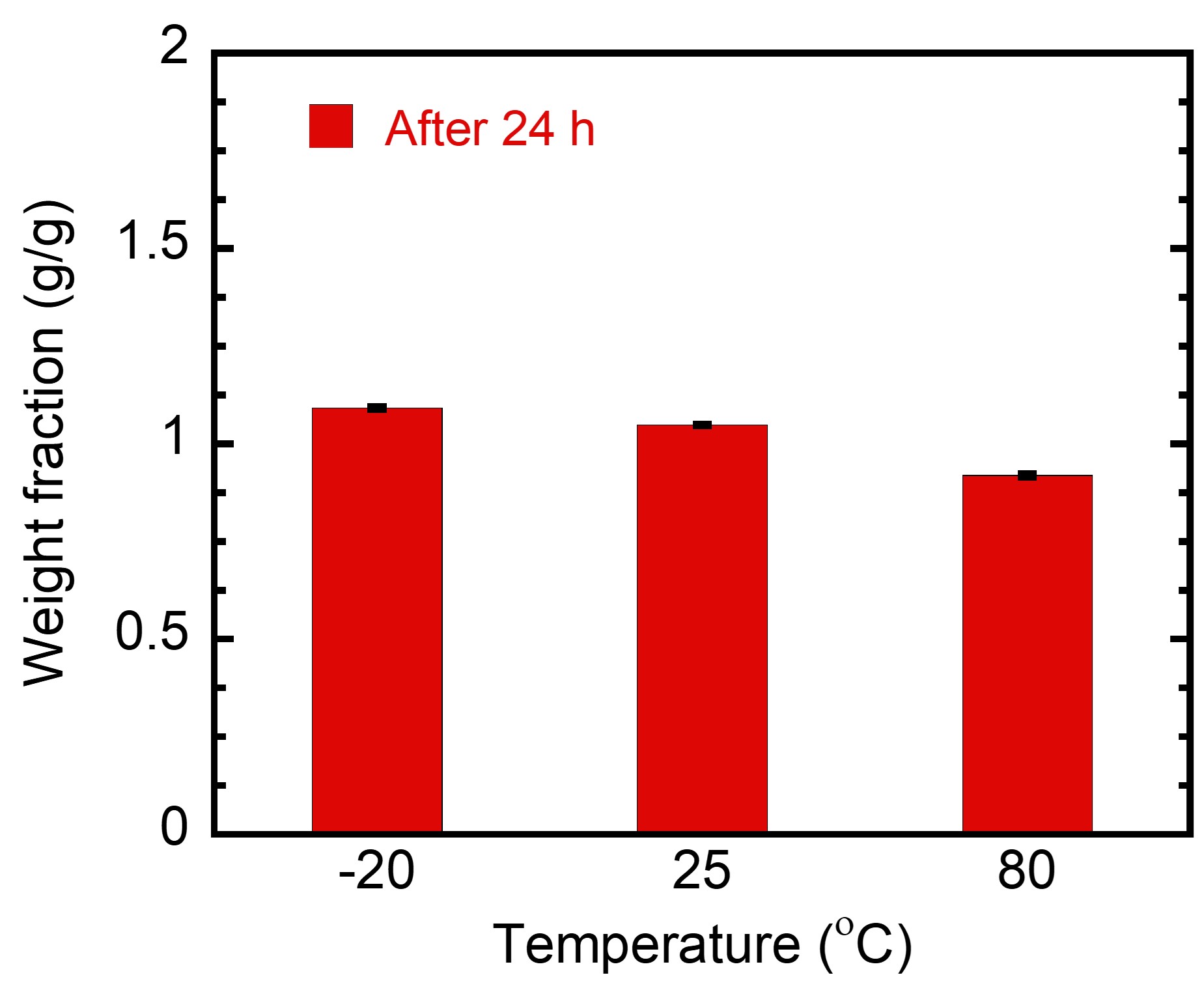


**Figure S15.** Weight change of AGSC after incubating the device at –20, 25, and 80 °C for 24 h. Data are presented as the mean value with absolute deviations (*n* = 3).


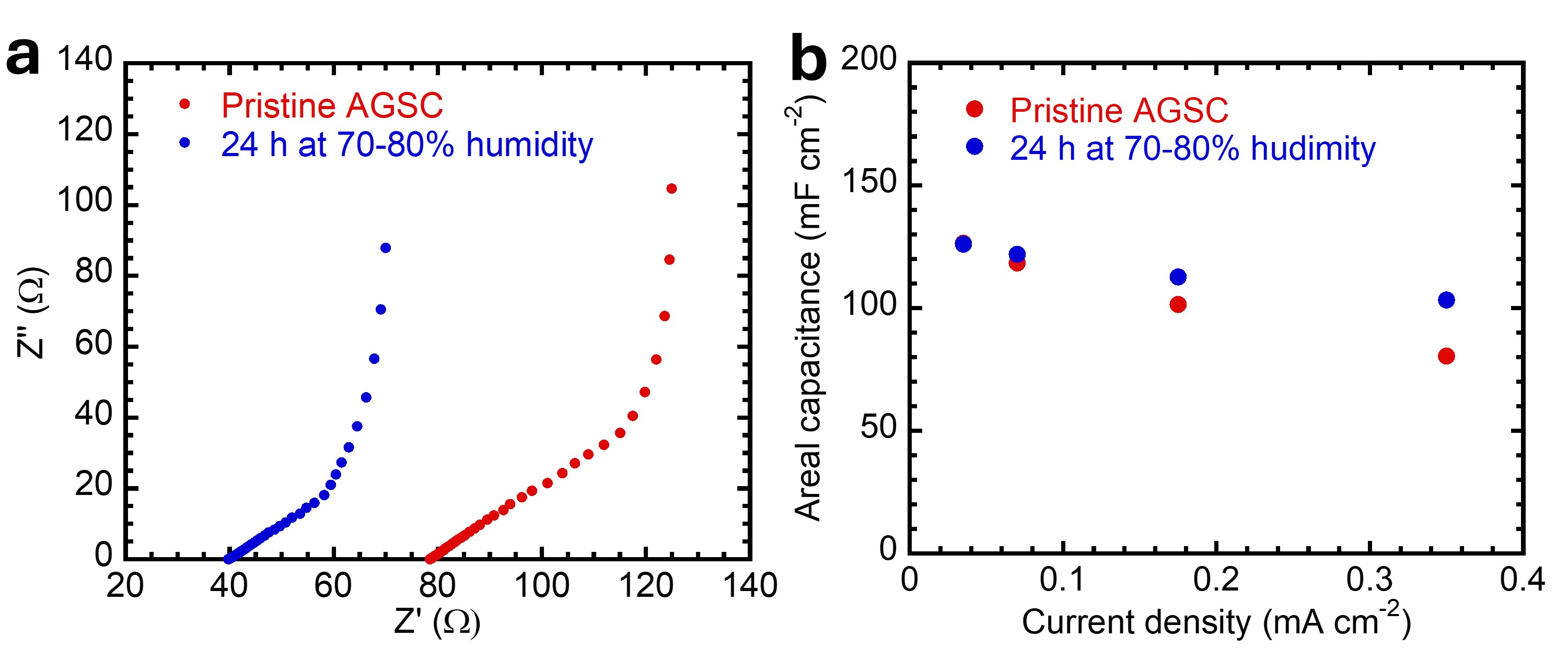


**Figure S16.** (a) Nyquist curves and (b) areal capacitance as a function of the current density for pristine AGSC and after equilibrating the device at 70%–80% humidity for 24 h.

**Table S1** Comparison of the areal capacitance and capacitance retention of the reconfigurable AGSC with those of other reported state-of-the-art AGSCs.

| Reference | Capacitance @ current density/scan rate | Capacitance retention (%) | Number of charge-discharge cycles @ current density/scan rate |
| --- | --- | --- | --- |
| E-SC^[16]^ | 24.90 mF cm^-2^ @ 0.5 mA cm^−2^ | 90.60 | 10,000 @ 0.5 mA cm^-2^ |
| AF-SSC^[17]^ | 14.40 mF cm^-2^ @ 0.03 mA cm^−2^ | 90.00 | 10,000 @ 100 mV s^-1^ |
| PANI-SC^[18]^ | 137.40 mF cm^-2^ @ 0.5 mA cm^−2^ | 82.00 | 2,000 @ 2 mA cm^-2^ |
| SWCNT/PANI/PVA-SC^[19]^ | 15.80 mF cm^-2^ @ 0.044 mA cm^−2^ | 88.00 | 2,000 @ 0.13 mA cm^-2^ |
| BB-MSC^[20]^ | 251 mF cm^−2^ @ 0.1 mA cm^−2^ | 80.00 | 10,000 @ 4 mA cm^-2^ |
| SPGL-SC^[21]^ | 110.8 mF cm^−2^ @ 0.2 mA cm^−2^ | 88.50 | 10,000 @ 1 mA cm^-2^ |
| PANI/PVA-SC^[22]^ | 166 mF cm^−2^ @ 0.5 mA cm^−2^ | 87.00 | 3,000 @ 4 A g^-1^ |
| **This work** | **449.85 mF cm^−2^ @ 0.035 mA cm^−2^** | **88.95**  **100.0** | **20,000 @ 0.35 mA cm^-2^**  **10,000 @ 0.35 mA cm^-2^** |
